# Analysis of Short-Term Responses to Hypoxia During Stirred-Tank Fermentation in *Aspergillus oryzae*

**DOI:** 10.3390/jof12050347

**Published:** 2026-05-07

**Authors:** Soma Araki, Shunya Susukida, Ken Miyazawa, Toshitaka Kumagai, Jikian Tokashiki, Keietsu Abe

**Affiliations:** 1 Major of Agricultural Chemistry Graduate School of Agricultural Science, Tohoku University, Sendai 980-8572, Japan; soma.araki.r3@dc.tohoku.ac.jp (S.A.); shunya.susukida.p2@dc.tohoku.ac.jp (S.S.); miyazawa.ke@jihs.go.jp (K.M.); jikian.tokashiki.b2@tohoku.ac.jp (J.T.); 2 Fermlab Inc., Tokyo 135-0021, Japan; tk@fermlab.com

**Keywords:** hypoxia, bioreactor, *Aspergillus oryzae*, multi-omics, respiratory chain, alternative oxidase, ROS

## Abstract

During fermentation in stirred-tank bioreactors (STBR), filamentous fungi are frequently exposed to hypoxic conditions. However, their responses, especially short-term ones (≤6 h), remain unclear. In this study, we performed a short-term multi-omics profiling in an *Aspergillus oryzae* hyphal dispersion mutant (AGΔ-GAGΔ) during a controlled transition to hypoxia (a decrease in dissolved oxygen (DO) from 10% to ≤1%) in a 4 L STBR. In transcriptome analysis, the genes encoding mitochondrial respiratory chain Complexes I–III were transiently downregulated at 1 h from DO depletion and were then upregulated, whereas those of Complex IV were upregulated immediately at the onset of hypoxia. In relation to this respiratory remodeling, we also observed an immediate induction of an alternative oxidase (AOX) gene. However, our metabolome data showed no significant change in the ATP level. This result could be explained by the upregulation of the glycolytic genes in hypoxic cultures. Fluorescence imaging revealed a transient increase in intracellular reactive oxygen species (ROS) in hypoxia, and metabolomics data revealed a decrease in the reduced glutathione/oxidized glutathione ratio in hypoxic cultures. Deletion of the AOX gene prolonged the ROS increase. Together, these data indicate that early hypoxia triggers a transient increase in oxidative stress, mitigated by antioxidant systems and mitochondrial respiratory rebalancing including an AOX-mediated bypass.

## 1. Introduction

Filamentous fungi are widely used for production of diverse bioactive and industrially relevant organic molecules [1,2]. In industrial applications, stirred-tank bioreactor (STBR) fermentation is common as it provides efficient oxygen transfer to meet the high oxygen demand of filamentous fungi [3,4]. During cultivation, the viscosity of the culture broth increases due to high biomass and characteristic morphology (e.g., hyphal branching, entanglement and agglomeration), and oxygen transfer becomes limiting, leading to hypoxic environments despite continuous aeration [5].

*Aspergillus oryzae* is widely used as a generally recognized as safe (GRAS) production host and meets diverse industrial needs in food fermentation and food-enzyme manufacturing [6]. We previously reported that cell-wall engineering by deletion of α-1,3-glucan (AG) and galactosaminogalactan (GAG) (AGΔ-GAGΔ) improved hyphal dispersion and less macroscopic aggregation in *A. oryzae* [7]. In this genetic background, viscosity decreased and mass transfer in liquid culture was enhanced, while the capacity to secrete recombinant enzymes was improved [8,9]. However, even for such strains, dynamic drops in dissolved oxygen (DO) were common as the culture density increased [9].

Several omics studies have sought to provide a comprehensive overview of hypoxic responses in filamentous fungi [10,11,12], reporting a wide range of transcriptomic and metabolomic changes. These changes relate to biomass growth and, in some cases, industrial enzyme production [13]. Most works have focused on long-term (>6 h) comparisons [10,13] or shake-flask/plate systems [12], even though oxygen limitation can trigger rapid transcriptional changes and stress responses in fungi [14,15]. In contrast, omics studies under controlled STBR culture conditions remain scarce [16], particularly those of short-term responses (≤6 h).

Some studies have reported that early hypoxia elicits a coordinated program that rebalances mitochondrial respiratory flux, redox balance, and cofactor supply [16,17,18]. Hypoxia decreases electron acceptor availability and can increase electron accumulation within the electron transport chain (ETC) [19]; these effects are accompanied by an increase in intracellular ROS that can lead to mitochondrial oxidative damage [20,21]. To counteract such oxidative stress, not only filamentous fungi but also other fungi and plants induce antioxidant systems and can engage the alternative oxidase (AOX) branch of mitochondrial respiration as a bypass that relieves electron congestion at the ubiquinone pool, thereby helping mitigate ROS generation when cytochrome pathway flux is limited [17,21,22,23]. Even though extensive studies have been conducted to understand hypoxic responses in various organisms, they have not been examined in detail in STBR fermentation of filamentous fungi including *A. oryzae* [12] during short-term hypoxia (≤6 h).

The aim of this study was to resolve the short-term (≤6 h) hypoxic responses of *A. oryzae* during fermentation in an STBR. We imposed a controlled transition to hypoxia in an STBR and assessed the transcriptional and metabolomic responses as well as reactive oxygen species (ROS) accumulation. To investigate how the ETC is transcriptionally rebalanced and how energy homeostasis and redox balance are sustained, we integrated time-resolved transcriptomics and metabolomics. We also assessed whether hypoxia perturbs intracellular ROS and mitochondrial state using fluorescence imaging. To specifically probe the contribution of AOX, we produced a Δ*aoxA* strain of *A. oryzae*, which lacks the predominant AOX paralog *aoxA* in the AGΔ-GAGΔ background, and evaluated this strain under hypoxic conditions. This allowed us to test whether an AOX-mediated respiratory bypass underlies acclimation during early hypoxia in STBR culture.

## 2. Materials and Methods

### 2.1. Strains and Inoculum Preparation

*Aspergillus oryzae* WT-cutL1 (WT) was used as our control strain [24]. The *A*. *oryzae* α-1,3-glucan synthase gene and galactosaminogalactan synthase gene knockout mutant AGΔ-GAGΔ-cutL1 (AGΔ-GAGΔ) were used to minimize hyphal aggregation [7]. The AGΔ-GAGΔ-ΔAOXA-cutL1 (AGΔ-GAGΔ-ΔAOXA) strain derived from AGΔ-GAGΔ-cutL1 was constructed following the protocol described in Miyazawa et al. (2019) [7] (Tables S1 and S2). These strains were maintained on Czapek–Dox agar plates for 7 days and then on malt agar plates for 7 days to obtain spores. Conidia for inoculation into bioreactors were prepared by isolating spores in phosphate-buffered saline (PBS) containing 0.1% Tween-20 by filtering through Miracloth (Merck Millipore, Darmstadt, Germany).

### 2.2. Cultivation in a Lab-Scale Bioreactor

**Figure 1:**
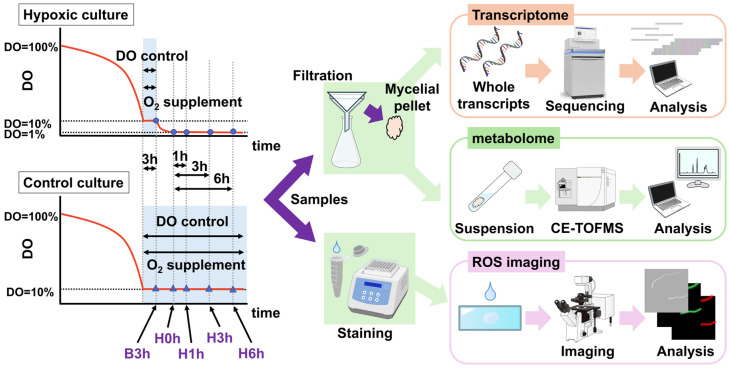
Dissolved oxygen-controlled cultivation and multi-omics workflow in the AGΔ-GAGΔ strain of *Aspergillus oryzae*. DO, dissolved oxygen; ROS, reactive oxygen species; CE-TOFMS, capillary electrophoresis–time-of-flight mass spectrometry.

The fermenter used was a 4 L bioreactor HSF-3000 with an HS100 turbine and an HR100 impeller (all from Satake Multimix Corporation, Saitama, Japan), equipped with other devices as described in Susukida et al. (2025) [9] (Figure S1). For multi-omics, modified chemically defined medium (Table S3) and the DO were saturated by aeration with ambient air at 30 °C before inoculum. An initial conidia concentration of 5 × 10^5^/mL was used. The initial culture volume was 2.1 L, and the aeration was 1.05 L/min (0.5 vvm [volume of gas per volume of liquid per minute]) for the first 24 h (30 h for WT) and 1 vvm afterwards. Cultures were incubated at 30 °C with agitation at 300 rpm for 24 h (30 h for WT) and then at 700 rpm until 60 h. The DO regime is depicted in Figure 1. When DO first reached 10%, the supply of feed medium containing 50% glucose and 4% urea was started at 0.15 mL/min until the end of fermentation. From the same timepoint, the DO was maintained at 10% for 3 h by automatically changing the ratio of ambient air and pure oxygen gas while maintaining total flow rate. After 3 h of controlling DO, it was maintained at 10% as long as possible in the control cultures, whereas the hypoxic culture was aerated with ambient air (without any additional 100% oxygen gas).

### 2.3. Experimental Design and Multi-Omics Workflow

For both control and hypoxic cultures, the timepoint after 3 h of maintaining the DO level at 10% was designated as Baseline 3 h (B3h). The time at which the DO first reached 1% in the hypoxic culture (Figure 1) was designated as Hypoxia 0 h (H0h), and 1 h, 3 h, and 6 h after H0h as H1h, H3h, and H6h, respectively. For the control culture, H0h was based on the time frame of the hypoxic culture (Figure 1). Actual sampling timepoints for all cultures and replicates are shown in Figure S2 (note that all samples were taken under constant agitation and aeration rates). For transcriptome analysis, WT, AGΔ-GAGΔ, and AGΔ-GAGΔ-ΔAOXA were used. For metabolome analysis, AGΔ-GAGΔ was used. For ROS assay and mitochondrial staining, AGΔ-GAGΔ and AGΔ-GAGΔ-ΔAOXA were used. Note that only hypoxic culture was conducted for AGΔ-GAGΔ-ΔAOXA.

### 2.4. Sample Preparation for RNA-Seq

Mycelia were collected by filtration, immediately frozen in liquid nitrogen and kept at −80 °C until use. The mycelia were homogenized in liquid nitrogen, and total RNA was extracted with a NucleoSpin RNA Plus kit (Macherey-Nagel GmbH & Co. KG, Düren, Germany; as per the manufacturer’s instructions). The mRNA libraries were then generated from total RNA with an Oligotex-dT30 mRNA Purification Kit (Takara Bio Inc., Kusatsu, Shiga, Japan; as per the manufacturer’s protocol) and sequenced on the Illumina platform by Macrogen Japan Corp. (Tokyo, Japan). Three biological replicates per condition were used for WT and AGΔ-GAGΔ-ΔAOXA, and four for AGΔ-GAGΔ.

### 2.5. Transcriptome Analysis

The generated fastq files were processed in fastp [25] to remove low-quality reads and adapters. All passed reads were mapped to *A. oryzae* RIB40 (GCF_000184455) using STAR [26]. Raw transcript count and normalization, as well as gene annotation were performed using FeatureCounts [27]. The Gene Transfer Format file we used is a custom file fixing several errors in the existing gene models of *A. oryzae* RIB40 genome annotation, available on GitHub (https://github.com/Fermentation-Lab/database_rna-seq, accessed on 1 March 2026).

### 2.6. Gene Ontology (GO) Enrichment Analysis

Analysis was performed on the differentially expressed genes (DEGs) identified in the transcriptome analysis for each strain, timepoint, and DO condition [28,29]. All DEGs for each strain and condition between any two timepoints were used in a clustering analysis of gene expression patterns. Z-score-standardized log_10_TPM was calculated for each sample and gene across the timepoints, and those z-score vectors were grouped into 20 clusters using an unsupervised k-means method. Genes for which 75% or more of biological replicates were allocated to a certain cluster were assigned to this cluster. To identify the sets of genes with consistent expression patterns according to timepoints, GO enrichment analysis was performed on each cluster. Gene sets with FDR  <  0.05 were considered significantly enriched in a cluster.

### 2.7. Metabolome Analysis

Mycelia were prepared in three biological replicates as in the protocol supplied by Human Metabolome Technologies (Yamagata, Japan) [9], and the samples were subjected to capillary electrophoresis–time-of-flight mass spectrometry (CE-TOFMS) performed by Human Metabolome Technologies. Measured values were normalized by the wet mycelial weight. For statistical analysis, chromatographic peak areas normalized to those of the internal standard and to biomass (normalized abundance) were treated as missing when reported as “not detected”; no imputation was performed. To stabilize variance and accommodate zeros, normalized abundances were log-transformed using a per-metabolite pseudocount ε, defined as one-half of the smallest positive value (fallback ε = 1 × 10^−12^ if none). For each metabolite we fit a linear mixed-effects model with DO status (control vs. hypoxic) and sampling time (B3h–H6h) as fixed effects and a random intercept for the biological replicate; models were estimated by maximum likelihood. Global effects (DO, time, interaction) were assessed by likelihood-ratio tests against reduced models. Two a priori comparisons were made using data on the log scale: hypoxic vs. control at each timepoint, and change from B3h within each condition. Fixed-effect coefficients on the natural-log scale (β_ln) are reported, where β_ln is the estimated difference between conditions. Two-sided *p*-values were Benjamini–Hochberg-adjusted within each test family across metabolites. To examine redox balance, the NADH and NAD^+^, NADPH and NADP^+^, and reduced glutathione (GSH) and oxidized glutathione (GSSG) pairs were analyzed using log-ratios ln(x_1_ + ε_1_) − ln(x_2_ + ε_2_) and modeled with the same mixed-effects design and contrast scheme; x_1_ and x_2_ are the within-sample normalized abundances of the reduced and oxidized species, respectively, and ε_1_ and ε_2_ are the corresponding pseudocounts. Analyses were performed in Python 3.12.12. Three biological replicates per condition were used.

### 2.8. ROS Assay and Mitochondrial Staining

Culture aliquots (20 μL) were mixed with 10 μM ROS Assay Kit-Photo-oxidation Resistant DCFH-DA (R253, Dojindo, Kumamoto, Japan) and 250 nM MitoTracker Deep Red (Thermo Fisher Scientific, Waltham, MA, USA), and incubated for 15 min at 30 °C. Mycelia were fixed in 4% paraformaldehyde in PBS with 10% glycerol and examined under a fluorescence microscope (IX-71, Olympus, Tokyo, Japan). The fluorescence signals of 2′,7′-dichlorodihydrofluorescein diacetate (DCFH-DA) and MitoTracker Deep Red were detected. Differential interference contrast (DIC) images were taken for each capture at the same time. Three biological replicates were prepared for each DO condition and timepoint, and one field of view per replicate was processed. Contrast and brightness in all captured 16-bit images were adjusted in the same way in Fiji [30]. In each adjusted image, the pixels in hyphae were filtered by applying the threshold value obtained by Otsu’s method [31], and fluorescence intensities in those pixels were measured. The Pearson correlation coefficient between the raw dichlorofluorescein (DCF) and MitoTracker Deep Red fluorescence intensities was computed in Python.

## 3. Results

### 3.1. Global Short-Term Transcriptional Responses to Hypoxia in WT and AGΔ-GAGΔ Strains

To assess the short-term hypoxic responses of *A. oryzae* in STBR fermentation, we performed RNA-seq in WT, AGΔ-GAGΔ, and AGΔ-GAGΔ-ΔAOXA at controlled DO concentrations. The actual DO data are shown in Figure S3. Hierarchical clustering showed good reproducibility among the biological replicates (Figure S4). GO term enrichment was analyzed on DEGs (FDR < 0.05) between any two timepoints within each DO treatment and between the corresponding timepoints across different DO treatments. The numbers of DEGs in each strain are shown in Tables S4 and S5, and the numbers of enriched GO terms in Table S6. Overviews of the enrichment of each GO term in the WT and AGΔ-GAGΔ strains are shown in Figures S5 and S6.

Hereafter, we mainly focused on AGΔ-GAGΔ as it had more biological replicates (*n* = 4) than WT (*n* = 3) or AGΔ-GAGΔ-ΔAOXA (*n* = 3) and fewer hyphal aggregation effects than WT.

**Figure 2:**
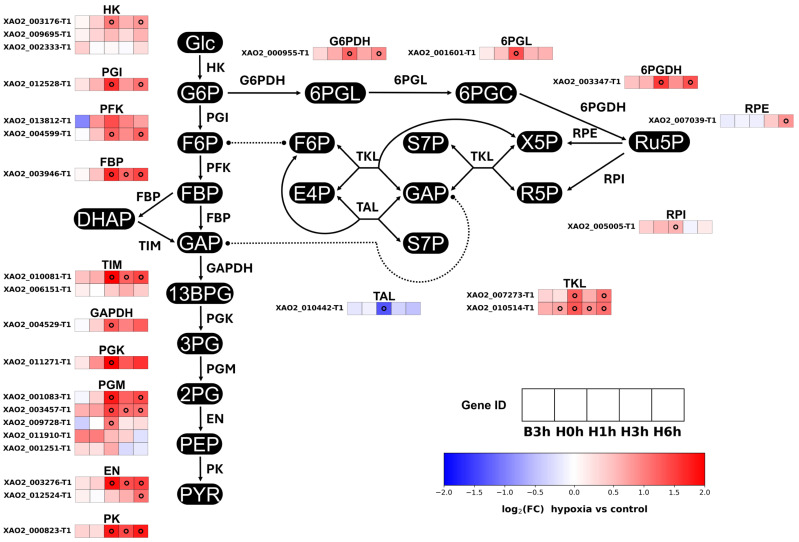
Expression of glycolysis and pentose phosphate pathway genes in control and hypoxic cultures of AGΔ-GAGΔ. Columns show sampling timepoints, rows show paralogs and their gene IDs. Red, high expression in hypoxic condition; blue, high expression in control condition. For ≥2 paralogs, upper rows represent higher expression levels, sorted on the basis of average TPM values across times, replicates and conditions. Black circles denote false discovery rate (FDR) < 0.05 (hypoxic vs. control). Metabolites: Glc, glucose; G6P, glucose-6-phosphate; F6P, fructose-6-phosphate; FBP, fructose-1,6-bisphosphate; DHAP, dihydroxyacetone phosphate; GAP, glyceraldehyde-3-phosphate; 13BPG, 1,3-bisphosphoglycerate; 3PG, 3-phosphoglycerate; 2PG, 2-phosphoglycerate; PEP, phosphoenolpyruvate; PYR, pyruvate; 6PGL, 6-phosphogluconolactone; 6PGC, 6-phosphogluconate; Ru5P, ribulose-5-phosphate; R5P, ribose-5-phosphate; X5P, xylulose-5-phosphate; S7P, sedoheptulose-7-phosphate; E4P, erythrose-4-phosphate. Enzymes: HK, hexokinase; PGI, phosphoglucose isomerase; PFK, phosphofructokinase; FBP, fructose-bisphosphate aldolase; TIM, triosephosphate isomerase; GAPDH, glyceraldehyde-3-phosphate dehydrogenase; PGK, phosphoglycerate kinase; PGM, phosphoglycerate mutase; EN, enolase; PK, pyruvate kinase; G6PDH, glucose-6-phosphate dehydrogenase; 6PGL, 6-phosphogluconolactonase; 6PGDH, 6-phosphogluconate dehydrogenase; RPI, ribose-5-phosphate isomerase; RPE, ribulose-5-phosphate epimerase; TKL, transketolase; TAL, transaldolase.

In the AGΔ-GAGΔ strain, GO terms were significantly enriched for pathways such as glycolysis, pentose phosphate pathway (PPP), heme biosynthesis, RNA processes, and redox processes (Figure S6). Enrichment was widely observed in the nucleus, cytoplasm, and mitochondrion (Figure S6). Genes for glycolysis and PPP enzymes were upregulated at H1h, and their expression remained high through H6h (Figure 2). Downstream of PPP, some genes involved in nucleotide synthesis, degradation, or interconversion were upregulated (most prominently at H1h) in hypoxic culture (Table S7). We also detected rRNA- and tRNA-related DEGs (Figure S6). Expression of genes for antioxidant enzymes such as peroxiredoxins and catalases increased at different timepoints; the strongest increase was observed for peroxiredoxins at H1h (Table S7). Some genes related to heme synthesis (conversion of 4 porphobilinogen into protoporphyrinogen IX) were also upregulated at H1h (Table S7). Coproporphyrinogen-III oxidase, the first enzyme that uses molecular oxygen in the heme biogenesis pathway, was particularly drastically upregulated under hypoxia (Figure S7). Within the glutathione system, the expression of genes for glutathione synthetase (GS), glutathione peroxidase (GPX), and glutathione reductase (GR) increased at H1h in the hypoxic culture relative to the control culture (Figure S7). Among those enzymes, the expression of the gene for GPX remained elevated at H6h relative to that in the control culture, whereas that of the other genes returned to the baseline at H3h (Figure S7).

Transcriptional changes were more pronounced in the hypoxic cultures than in the control cultures (Tables S4–S6), so we further analyzed the hypoxic cultures.

### 3.2. ETC-Focused Transcriptional Analysis

**Figure 3:**
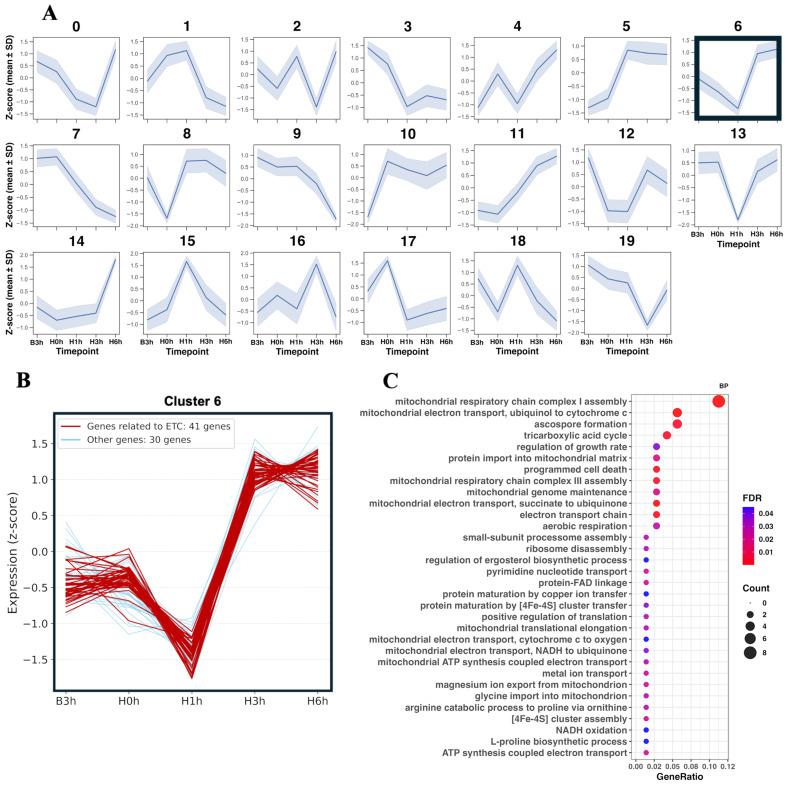
Clustering analysis based on gene expression patterns in AGΔ-GAGΔ hypoxic culture. (**A**) Twenty separate clusters. Solid lines, mean values of four biological replicates; shading, standard deviation. (**B**) Expression patterns of genes in cluster 6. Each line, an individual gene; red lines, genes related to the respiratory chain (ETC). (**C**) Gene Ontology (GO) terms in Biological Process enriched (false discovery rate (FDR) < 0.05) in cluster 6.

We next examined the time-dependent transcriptional responses in the AGΔ-GAGΔ hypoxic cultures. To extract the gene sets with similar expression patterns, we used clustering based on the expression patterns (Figure 3A). Among the top five clusters by gene count, cluster 6 was dominated by ETC-related genes; therefore, we examined it in detail (Figure 3B,C). In this cluster, transcript levels decreased temporarily at H1h and then increased from H3h to H6h above the B3h levels.

**Figure 4:**
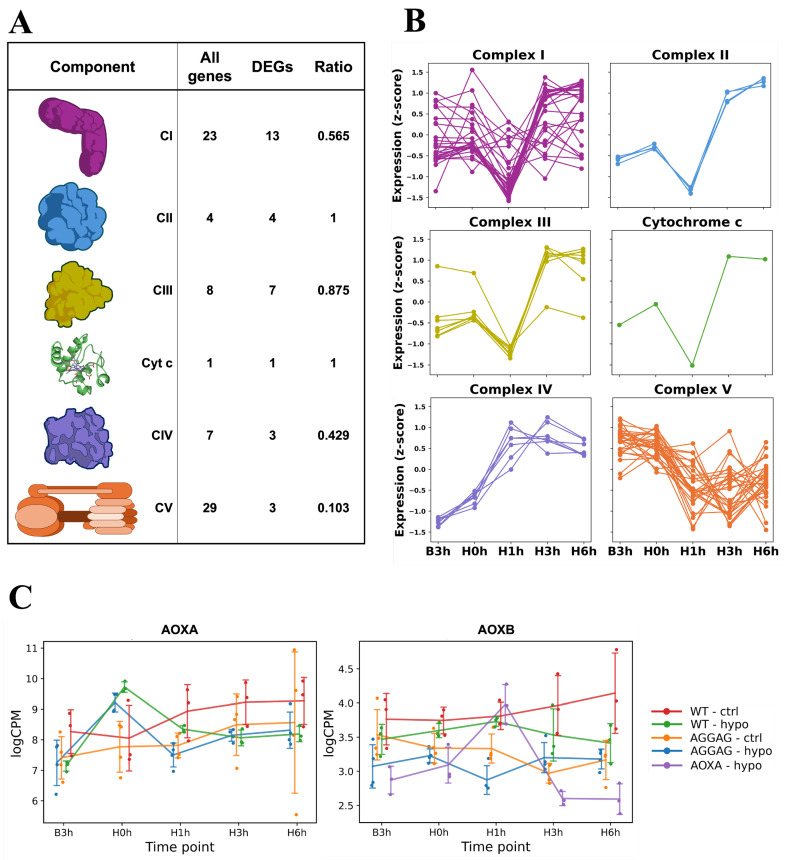
Expression of mitochondrial respiratory chain genes in AGΔ-GAGΔ hypoxic culture. (**A**) Numbers of differentially expressed genes (DEGs) and their ratios among all annotated genes for each. CI, Complex I; CII, Complex II; CIII, Complex III; Cyt c, Cytochrome *c*; CIV, Complex IV; CV, Complex V. (**B**) Z-score-standardized expression patterns of all genes for each component. (**C**) Expression patterns of AOX paralogs.

To compare the magnitude and timing of expression across genes for mitochondrial respiratory complexes, we grouped all genes annotated as encoding the components of Complexes I–V plus cytochrome *c* (annotation-based gene sets; Table S8) and plotted the expression patterns for each complex (Figure 4A,B). In the AGΔ-GAGΔ hypoxic culture, genes of Complexes I–III and cytochrome *c* had the same expression patterns as did the cluster shown in Figure 3B. In contrast, the expression of genes for Complex IV (cytochrome *c* oxidase) was already upregulated at H1h and remained elevated through H6h. For Complex V, only 3 genes out of 29 met the FDR < 0.05 criterion in the hypoxic vs. control culture comparisons at the same timepoints (Figure 4A,B). No such changes in the expression of ETC genes were observed in control cultures (Figure S8A). One of the AOX paralogs, *aoxA*, was immediately induced at H0h under hypoxia (log2FC = 1.88, FDR = 0.12 in AGΔ-GAGΔ; log2FC = 2.58, FDR = 0.04 in WT; both relative to B3h) and then declined at H1h (log2FC = −1.72, FDR = 0.03 in AGΔ-GAGΔ; log2FC = −1.65, FDR = 0.37 in WT; both relative to H0h), whereas the other paralog, *aoxB*, was not comparably induced in hypoxic cultures (Figure 4C).

To assess whether AOX affects the expression of genes coding for mitochondrial respiratory chain components in hypoxic cultures, we profiled the AGΔ-GAGΔ-ΔAOXA strain. Expression changes of Complex I–III genes were smaller in this strain, resulting in fewer DEGs for each complex (Figure S8A). Expression of the Complex IV genes still increased at H1h and remained elevated thereafter (Figure S8B). Most of the genes in Complex V were not detected as DEGs in AGΔ-GAGΔ-ΔAOXA. It is worth noting that *aoxB* expression transiently increased at H1h in AGΔ-GAGΔ-ΔAOXA (log2FC = 0.86, FDR = 0.04 for H1h vs. H0h; log2FC = −1.41, FDR = 0.004 for H3h vs. H1h), whereas it did not change significantly in AGΔ-GAGΔ or WT (Figure 4C).

In WT, the expression changes of genes for components of mitochondrial respiratory complexes were similar to those of AGΔ-GAGΔ-ΔAOXA rather than to those of AGΔ-GAGΔ (Figure S8A,B): DEGs were identified only in the genes for Complex IV and Complex V, and the Complex IV genes were significantly upregulated.

### 3.3. Imaging of Intracellular ROS and Mitochondria

**Figure 5:**
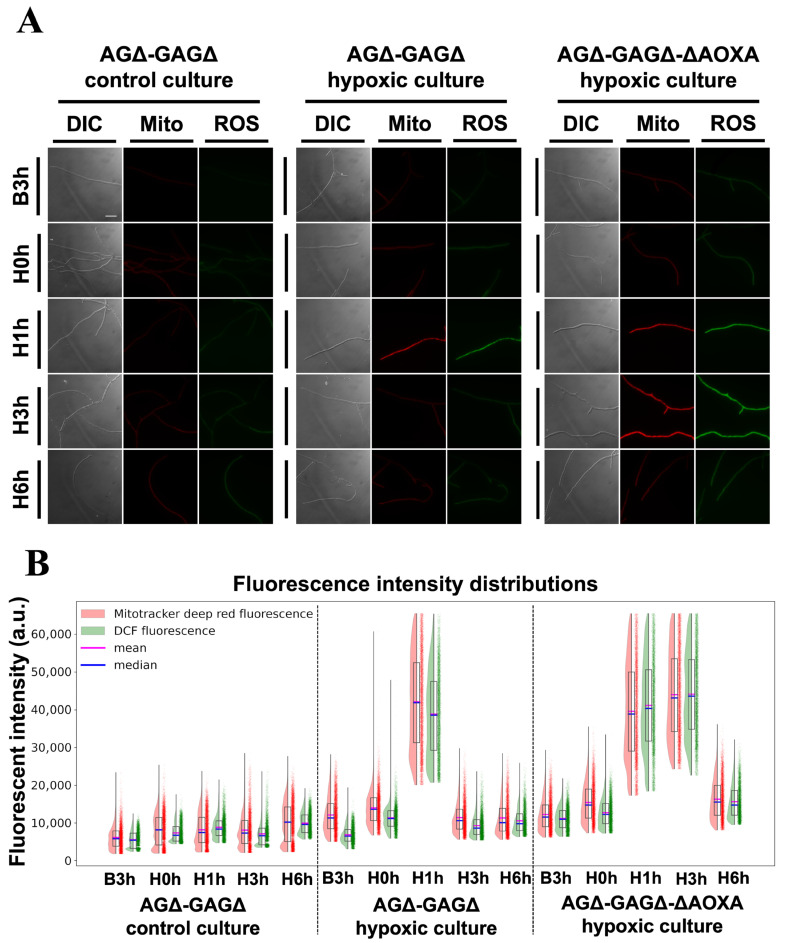
Fluorescence imaging in AGΔ-GAGΔ control and hypoxic cultures and AGΔ-GAGΔ-ΔAOXA hypoxic culture. (**A**) Representative images of the indicated cultures. DIC, differential interference contrast; Mito, MitoTracker Deep Red fluorescence; ROS, dichlorofluorescein (DCF) fluorescence. Scale bar = 20 μm. (**B**) Fluorescence intensity distributions in hyphae. For visualization, in total, 10,000 pixels in hyphae (equal number of pixels were randomly extracted from three biological replicates) were plotted on the right side of the half-violin plots for each sample. Median and mean values of fluorescence intensity of the pixels in hyphae are shown.

As the RNA-seq data highlighted the ETC, we qualitatively assessed ROS and mitochondrial state at the same five timepoints by fluorescence imaging. We stained intracellular ROS with the DCFH-DA dye and mitochondria with MitoTracker Deep Red in AGΔ-GAGΔ in both control and hypoxic cultures and in AGΔ-GAGΔ-ΔAOXA hypoxic culture (Figure 5). Only AGΔ-GAGΔ and AGΔ-GAGΔ-ΔAOXA were assessed because of uneven fluorescence intensity in WT. In AGΔ-GAGΔ, DCF fluorescence was low and stable in the control culture at all timepoints, but it transiently increased at H1h in the hypoxic culture and returned toward baseline at H3h. MitoTracker Deep Red also transiently increased at H1h in the hypoxic culture and returned to normal at H3h. In the AGΔ-GAGΔ-ΔAOXA hypoxic culture, DCF and MitoTracker signals also increased at H1h, remained elevated at H3h, and returned to the baseline at H6h. Raw intensities of DCF and MitoTracker Deep Red were positively correlated with each other (Pearson *r* = 0.992; Figure 5B and Figure S9).

### 3.4. Metabolome Analysis

**Figure 6:**
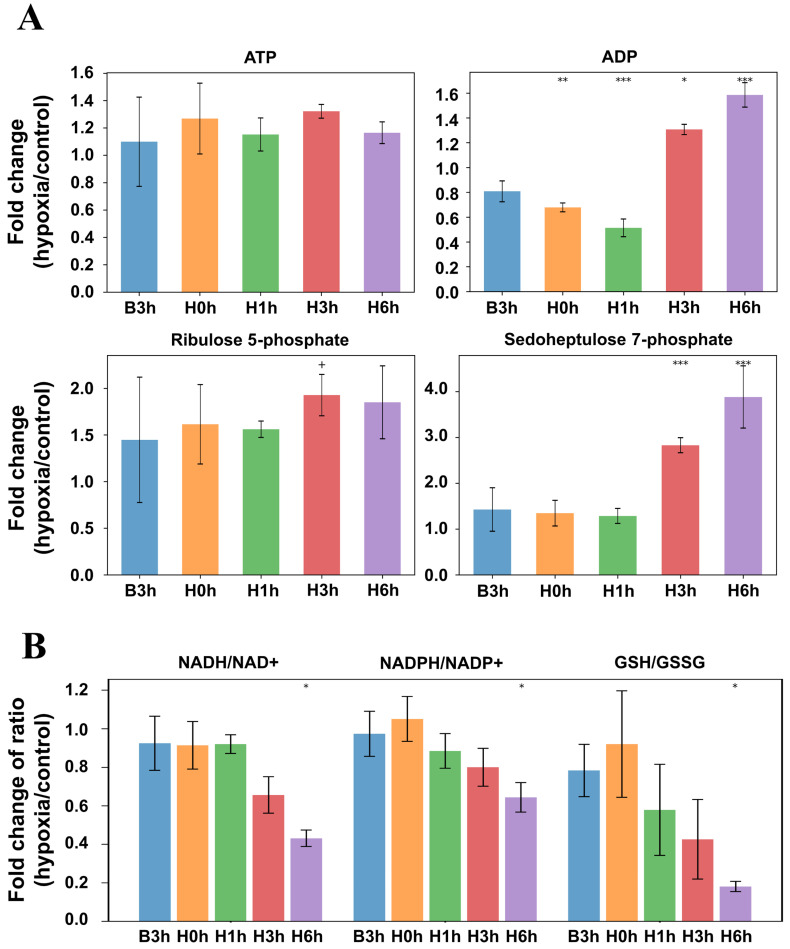
Relative fold differences in metabolites between hypoxic culture and control culture in AGΔ-GAGΔ. (**A**) Differences at each timepoint. (**B**) Ratios of the indicated metabolites at each timepoint. Comparisons vs. B3h: + *p* < 0.1, * *p* < 0.05, ** *p* < 0.01, *** *p* < 0.001.

As RNA-seq revealed hypoxia-dependent differences in the gene expression profiles for major metabolic pathways, including the ETC, we performed a metabolome analysis in AGΔ-GAGΔ. The results of principal component analysis are shown in Figure S10, and hierarchical clustering of metabolites is shown in Figure S11. Mixed-effects modeling detected a widespread interaction between conditions and timepoints. In the hypoxic vs. control culture comparisons at the same timepoints, ADP was significantly lower in the hypoxic culture at H1h and higher at H6h, whereas no changes were found in ATP levels (Figure 6A). In comparison to B3h within hypoxic culture, the ADP decrease at H1h was not significant (β_ln = −0.167, q = 0.166), but the increases at H3h (β_ln = +0.270, q = 4.7 × 10^−3^) and H6h (β_ln = +0.399, q = 5.2 × 10^−7^) were.

Among pyridine nucleotides, NADH progressively declined in the hypoxic culture, and the difference vs. B3h became significant at H6h, whereas NAD^+^ remained unchanged (Figure S12). Consistent with these changes, the NADH/NAD^+^ log-ratio tended to decrease at H3h and decreased significantly at H6h (Figure 6B). No significant changes were detected in the NADPH or NADP^+^ (Figure S12), but the NADPH/NADP^+^ log-ratio tended to decrease, with a significant decrease at H6h (Figure 6B). GSH was significantly lower at H6h vs. B3h, without significant changes in GSSG (Figure S12). The GSH/GSSG log-ratio decreased significantly at H6h (Figure 6B).

Among hypoxia-related intermediates, the level of sedoheptulose-7-phosphate was elevated vs. B3h at H3h and H6h, whereas that of ribulose 5-phosphate was not significantly affected at any timepoint (Figure 6A).

## 4. Discussion

In this study, we generated data on the short-term cellular responses of an *A. oryzae* hyphal-dispersion strain cultured in an STBR during a DO transition from 10% to ≤1%, combining time-course analyses of the transcriptome, targeted metabolomics, and fluorescence imaging of ROS and mitochondria (Figure 1).

According to the transcriptome analysis, genes involved in the PPP were significantly upregulated at H1h–H6h (Figure 2). Our metabolome analysis showed that sedoheptulose 7-phosphate, a PPP intermediate, accumulated significantly at H3h and H6h in hypoxic cultures, but another PPP intermediate, ribulose 5-phosphate (a precursor interconverted with ribose-5-phosphate in nucleotide synthesis), was not significantly affected (Figure 6A), whereas the NADPH/NADP^+^ log-ratio decreased at H6h with no significant changes in the NADPH or NADP^+^ pools (Figure 6B). PPP provides precursors for nucleotide and amino acid biosynthesis, and supplies NADPH for thiol maintenance and biosynthetic redox reactions via the PPP oxidative branch [32]. Understanding of the relationship between PPP and hypoxia remains limited. A proteomic study in *A. nidulans* in hypoxia reported an increased abundance of PPP enzymes and signatures of enhanced nucleotide turnover [33], whereas studies in other fungi reported either upregulation or downregulation at the transcriptional, flux, or protein levels [16,34,35]. The upregulation of most PPP genes in hypoxia in our present study might reflect potentially increased capacity for PPP flux. The downregulation of the transaldolase gene (Figure 2) could contribute to sedoheptulose 7-phosphate accumulation, as observed in humans with transaldolase deficiency [36]. Given the significant enrichment of rRNA- and tRNA-related DEGs in AGΔ-GAGΔ hypoxic cultures (Figure S6) and the upregulation of the genes involved in nucleotide synthesis, degradation, or interconversion (Table S7), the demand for nucleotides may increase in hypoxia, as reported by Shimizu et al. (2009) [33]; the maintained ribulose 5-phosphate pool could still be explained even if the capacity for PPP flux increases. Despite the upregulation of PPP oxidative-branch genes, the NADPH/NADP^+^ log-ratio decreased significantly at H6h, consistent with increased NADPH utilization in hypoxia, e.g., by the glutathione system to deal with oxidative stress [37].

Expression of genes encoding mitochondrial respiratory chain Complex IV increased immediately after the onset of hypoxia, while that of Complexes I–III was transiently downregulated at H1h in AGΔ-GAGΔ hypoxic cultures (Figure 4B). At the later timepoints (H3h and H6h), gene expression of these respiratory complexes was increased relative to B3h (Figure 3 and Figure 4B). The rapid induction of Complex IV is compatible with the enhanced terminal oxidase capacity in hypoxia, potentially improving electron throughput when oxygen availability is restricted. The gene expression increase in respiratory complexes at H3h and H6h is consistent with proteomic evidence of increases in mitochondrial respiratory complexes in hypoxia in *Aspergillus fumigatus* [38]. The expression of some heme-biosynthetic genes increased at H1h in hypoxic cultures (Figure S7, Table S7). Because these complexes are heme/iron-sulfur cluster-dependent, hypoxia often coincides with an increase in heme/iron-related pathways’ transcripts to sustain respiratory capacity [39,40,41]. Given these data, *A. oryzae* rapidly increases cofactor supply and terminal oxidase capacity after the onset of hypoxia. Consistent with this, the NADH/NAD^+^ log-ratio declined at H3h–H6h (driven by lower NADH) (Figure 6). These profiles are compatible with increased NADH oxidation via restored Complex I–IV flux, but decreased NADH production or cytosolic reoxidation may also contribute, so this inference remains provisional.

Hypoxia frequently elevates mitochondrial oxidant signals, classically via electron leak at Complex III and, when mitochondrial membrane potential (ΔΨ_m_) is high, via reverse electron transport (RET) at Complex I [42,43,44,45]. In AGΔ-GAGΔ hypoxic but not control cultures, DCF fluorescence and MitoTracker Deep Red increased at H1h and returned to the baseline at H3h (Figure 5). Although we did not measure the coenzyme Q (CoQ)/CoQH_2_ ratio or RET directly, the coordinated DCF and MitoTracker Deep Red transients are compatible with a transient shift toward a more reduced CoQ redox state and increased ΔΨ_m_, given the ΔΨ_m_-dependence of MitoTracker Deep Red fluorescence [46]. This transient ROS and MitoTracker peak at H1h coincided with transient downregulation of Complexes I–III (Figure 3 and Figure 4), implying that suppression of upstream ETC flux might temporarily limit excessive electron pressure at the CoQ pool and mitochondrial ROS formation. Many organisms recruit AOX to mitigate such CoQH_2_ pool over-reduction [47,48,49]. Hypoxia-induced AOX activity decreases mitochondrial ROS in multiple species [50,51,52,53]. In *A. oryzae*, AOX is conserved but incompletely characterized [22]. In AGΔ-GAGΔ and WT hypoxic cultures, *aoxA* was immediately upregulated at H0h, and returned toward baseline at H1h (Figure 4C). With the deletion of *aoxA*, the DCF and MitoTracker fluorescence increased at H1h and H3h, normalizing at H6h (Figure 5). Taken together, the strong correlation between DCF and MitoTracker fluorescence, *aoxA* expression patterns, and the prolongation of the high-ROS period in AGΔ-GAGΔ-ΔAOXA suggests that AOX attenuates intracellular oxidant accumulation via a respiratory bypass in *A. oryzae* during STBR fermentation, in line with AOX-linked ROS mitigation described in other organisms such as human, mouse, and *A. fumigatus* [54,55,56]. In AGΔ-GAGΔ-ΔAOXA, *aoxB* was transiently induced at H1h only (Figure 4C). This induction indicates that *aoxB* might partially compensate for the lack of *aoxA*, considering the difference in the transient expression increase timing between them, as indicated by an evolutionary study [22]. The effects of AOX on oxidative-stress tolerance and growth in hypoxic cultures remain to be determined.

In addition to AOX bypass, some antioxidant systems might also help deal with high oxidative stress [21]. Genes encoding peroxiredoxins were transiently upregulated at H1h and those encoding catalases also upregulated in hypoxic cultures at later timepoints (H3h and H6h) (Table S7). We focused on the glutathione system, which detoxifies ROS [57,58]; GS, GR, and GPX all increased at H1h; GS and GR returned to the baseline at H3h, whereas GPX remained elevated until H6h (Figure S7). These patterns align with fluorescence imaging data, which showed a transient ROS increase at H1h (Figure 5), and with our metabolome data, which showed a decrease in the NADPH/NADP^+^ log-ratio at H6h (Figure 6B), consistent with the dependence of GR on NADPH [59,60] and its return of the gene expression at H3h. The sustained upregulation of GPX at H6h may reflect a continued need for ROS detoxification. Metabolome data corroborated these expression patterns: the GSH/GSSG log-ratio decreased at H6h due to a fall in GSH, whereas GSSG did not change significantly (Figure 6B), supporting transcriptional remodeling of the glutathione system under hypoxia. However, given that the oxidative stress decreased to the baseline at H6h according to our ROS assay, the cause of maintaining elevated GPX expression at this timepoint remains unclear. Taken together with the peroxiredoxin and catalase gene upregulation, these data indicate that cells lower ROS levels by enhancing antioxidant capacity as an adaptation when ROS levels increased in hypoxic cultures.

The expression of glycolytic genes increased significantly at H1h and remained elevated towards H6h in hypoxic cultures (Figure 2). Hypoxia upregulates glycolysis at transcriptional or protein levels in several fungi such as *Saccharomyces cerevisiae*, *Aspergillus nidulans* and *A. oryzae* [11,12,61]. Hypoxia often leads to the suppression of ATP generation through oxidative phosphorylation because of the insufficient terminal electron acceptors, so substrate-level phosphorylation may be enhanced [10,62]. Given these reports, the upregulation of glycolysis may contribute to ATP supply in hypoxia. Our metabolome data showed an unchanged ATP pool in hypoxic cultures despite a significant decrease in the ADP pool at H0h–H1h followed by a significant increase at H3h–H6h (Figure 6A); accordingly, the ATP/ADP log-ratio increased significantly at H0h–H1h, followed by a later decrease (Figure S12). The biphasic behavior of ADP and the ATP/ADP log-ratio indicates that cellular energy status was dynamically reorganized during adaptation to early hypoxia, even though the ATP pool remained unchanged. The upregulation of glycolysis or the dynamic respiratory remodeling may have contributed to the energy status. However, the energy status is influenced not only by ATP production but also by ATP consumption and broader metabolic reorganization, so this interpretation remains provisional.

Here, we propose an overall model of a response to early hypoxia in STBR fermentation in *A*. *oryzae*. Hypoxia constrains oxygen availability to the ETC. Electron flow may slow down, the redox state of the CoQ pool becomes more reduced, and ΔΨ_m_ may transiently rise—conditions that favor electron leak and ROS formation at specific sites of the mitochondrial respiratory chain (Complex III, and at high ΔΨ_m_, Complex I via RET). In our dataset, early downregulation of Complexes I–III followed by later upregulation implies that a transient dampening of ETC-derived ROS production might be achieved by increased proportion of the reduced form of CoQ and limiting excessive ΔΨ_m_, prior to the re-establishment of respiratory capacity. Concurrent upregulation of Complex IV likely enhances terminal oxidase capacity under hypoxia. Transient engagement of AOX provides an electron bypass from the CoQH_2_ pool, thereby limiting over-reduction and ROS generation rather than directly increasing O_2_ capture. In parallel, enhanced glycolysis supplies ATP via substrate-level phosphorylation, while antioxidant systems (e.g., glutathione, peroxiredoxins, and catalases) help maintain cellular redox balance.

## 5. Conclusions

We profiled short-term (≤6 h) hypoxic responses of *A. oryzae* during early hypoxia in a 4 L STBR by integrating transcriptomics, metabolomics, and fluorescence imaging approaches. Early hypoxia triggered a global remodeling of metabolism, including upregulation of glycolysis and the PPP, and a transient increase in oxidative stress. The AOX paralog *aoxA* was rapidly induced; its deletion prolonged the high-ROS period, indicating that AOX helps mitigate transient oxidant accumulation during acclimation. Overall, early hypoxia elicits an AOX-linked respiratory bypass alongside coordinated transcriptional and metabolic adaptations. This study provides comprehensive understanding of short-term hypoxic responses of *A. oryzae* in STBR fermentation and insights into potential strategies to improve industrial applications. For the latter purpose, future research integrating culture engineering with genetic and metabolic engineering will be required.

## Data Availability

The raw RNA-seq data are deposited in the DDBJ Sequence Read Archive under Bioproject ID PRJDB37930. The other data that support the findings of this study are available from the corresponding author upon reasonable request.

## Supplementary Materials

The following supporting information can be downloaded at: https://www.mdpi.com/article/10.3390/jof12050347/s1, Figure S1: Schematic diagram of the 4 L bioreactor; Figure S2: Actual sampling timepoints of the WT, AGΔ-GAGΔ, and AGΔ-GAGΔ-ΔAOXA cultures. AGGAG, AGΔ-GAGΔ; AOXA, AGΔ-GAGΔ-ΔAOXA; hypo, hypoxic culture; ctrl, control culture; Figure S3: Dissolved oxygen (DO) traces of the WT, AGΔ-GAGΔ, and AGΔ-GAGΔ-ΔAOXA cultures. Solid lines, mean values of three biological replicates; shading, standard deviation. AGGAG, AGΔ-GAGΔ; AOXA, AGΔ-GAGΔ-ΔAOXA; hypo, hypoxic culture; ctrl, control culture; Figure S4: Hierarchical clustering of RNA-seq samples across different timepoints. The dendrograms were constructed using the Ward linkage method based on the Euclidean distances between log2CPM values of genes of (A) WT, (B) AGΔ-GAGΔ, (C) AGΔ-GAGΔ-ΔAOXA. The y-axes represent the Euclidean distance. HYPO, hypoxic culture; CTRL, control culture; Figure S5: Gene Ontology (GO) term enrichment across timepoints in WT. Comparisons: hypoxia vs. control. GO terms with false discovery rate (FDR) < 0.05 are shown; no GO terms were enriched at B3h. BP, biological process; MF, molecular function; CC, cellular component; Figure S6: Gene Ontology (GO) enrichment across timepoints in AGΔ-GAGΔ. Comparisons: hypoxia vs. control. GO terms with false discovery rate (FDR) < 0.05 are shown. BP, biological process; MF, molecular function; CC, cellular component; Figure S7: Expression patterns of selected genes across timepoints in each strain and DO condition. CPOX, coproporphyrinogen III oxidase; GS, glutathione synthetase; GR, glutathione reductase; GPX, glutathione peroxidase. AGGAG, AGΔ-GAGΔ; AOXA, AGΔ-GAGΔ-ΔAOXA; hypo, hypoxic culture; ctrl, control culture; Figure S8: Expression of mitochondrial respiratory chain genes in AGΔ-GAGΔ control culture, AGΔ-GAGΔ-ΔAOXA hypoxic culture, and in WT hypoxic and control cultures. (A) Numbers of differentially expressed genes (DEGs) and their ratios among all annotated genes for each. Components whose ratios of DEGs were ≥0.5 are shown in red. CI, Complex I; CII, Complex II; CIII, Complex III; Cyt c, Cytochrome *c*; CIV, Complex IV; CV, Complex V. (B) Z-score–standardized gene expression patterns of the respiratory components whose ratios of DEGs were ≥ 0.5. Hypo, hypoxic culture; ctrl, control culture; Figure S9: Pixels selected by Otsu’s method. DIC, differential interference contrast; Mito, filtered pixels for MitoTracker Deep Red fluorescence; ROS, filtered pixels for dichlorofluorescein (DCF) fluorescence; Common, pixels filtered by applying a common threshold obtained by Otsu’s method to each sample. The same fields of view as in Figure 5 are shown. The Pearson correlation coefficient between the DCF and MitoTracker Deep Red fluorescence was calculated from the raw intensities of common pixels across all samples, and was 0.992. Scale bar = 20 μm; Figure S10: Principal component analysis of metabolite profiles. Each point represents an individual sample, colored by DO condition and timepoint. PC1 and PC2, the first and second principal components; percentage values in parentheses indicate the proportion of variance explained by each component. AGGAG, AGΔ-GAGΔ; hypo, hypoxic culture; ctrl, control culture; Figure S11: Hierarchical clustering of metabolite profiles. Each row represents a metabolite. Color intensity reflects relative metabolite abundance based on Z-score normalized values. AGGAG, AGΔ-GAGΔ; hypo, hypoxic culture; ctrl, control culture; Figure S12: Relative fold differences of metabolites between hypoxic culture and control culture of AGΔ-GAGΔ. (A) Differences at each timepoint. (B) Ratio of ATP/ADP at each timepoint. Comparisons vs. B3h: + *p* < 0.1, * *p* < 0.05, ** *p* < 0.01, *** *p* < 0.001. Values were normalized to control at each timepoint; Table S1: Strains used in this study; Table S2: Primers used for the construction of AGΔ-GAGΔ-ΔAOXA; Table S3: Composition of the modified chemically defined medium used for lab-scale fermentation; Table S4: Differentially expressed gene (DEG) counts in each strain. Conditions: CTRL, control culture; HYPO, hypoxic culture; Table S5: Differentially expressed gene (DEG) counts between strains. Conditions: CTRL, control culture; HYPO, hypoxic culture; Table S6: Gene set counts in each strain. Conditions: CTRL, control culture; HYPO, hypoxic culture; Table S7: Gene expression of selected genes; Table S8: Annotated gene sets for respiratory components.

## Abbreviations

The following abbreviations are used in this manuscript:

AG	α-1,3-glucan
AOX	alternative oxidase
CE-TOFMS	capillary electrophoresis time-of-flight mass spectrometry
CoQ	coenzyme Q (ubiquinone)
CPM	counts per million
DCF	dichlorofluorescein
DCFH-DA	2′,7′-dichlorodihydrofluorescein diacetate
DEGs	differentially expressed genes
DIC	differential interference contrast (microscopy)
DO	dissolved oxygen
ETC	electron transport chain
FDR	false discovery rate
GAG	galactosaminogalactan
GO	Gene Ontology
GPX	glutathione peroxidase
GR	glutathione reductase
GRAS	generally recognized as safe
GS	glutamine synthetase
GSH	reduced glutathione
GSSG	oxidized glutathione (glutathione disulfide)
FC	fold change
PBS	phosphate-buffered saline
PPP	pentose phosphate pathway
ROS	reactive oxygen species
SREBP	sterol regulatory element-binding protein
STBR	stirred-tank bioreactor
TPM	transcripts per million

## References

[1] Meyer V., Basenko E.Y., Benz J.P., Braus G.H., Caddick M.X., Csukai M., de Vries R.P., Endy D., Frisvad J.C., Gunde-Cimerman N. (2020). Growing a Circular Economy with Fungal Biotechnology: A White Paper. Fungal Biol. Biotechnol..

[2] Nevalainen H., Peterson R. (2014). Making Recombinant Proteins in Filamentous Fungi- Are We Expecting Too Much?. Front. Microbiol..

[3] Blakebrough N., Sambamurthy K. (1966). Mass Transfer and Mixing Rates in Fermentation Vessels. Biotechnol. Bioeng..

[4] Garcia-Ochoa F., Gomez E. (2009). Bioreactor Scale-up and Oxygen Transfer Rate in Microbial Processes: An Overview. Biotechnol. Adv..

[5] Garcia-Ochoa F., Gomez E., Santos V.E. (2020). Fluid Dynamic Conditions and Oxygen Availability Effects on Microbial Cultures in STBR: An Overview. Biochem. Eng. J..

[6] Kobayashi T., Abe K., Asai K., Gomi K., Juvvadi P.R., Kato M., Kitamoto K., Takeuchi M., Machida M. (2007). Genomics of *Aspergillus oryzae*. Biosci. Biotechnol. Biochem..

[7] Miyazawa K., Yoshimi A., Sano M., Tabata F., Sugahara A., Kasahara S., Koizumi A., Yano S., Nakajima T., Abe K. (2019). Both Galactosaminogalactan and α-1,3-Glucan Contribute to Aggregation of *Aspergillus oryzae* Hyphae in Liquid Culture. Front. Microbiol..

[8] Ichikawa H., Miyazawa K., Komeiji K., Susukida S., Zhang S., Muto K., Orita R., Takeuchi A., Kamachi Y., Hitosugi M. (2022). Improved Recombinant Protein Production in *Aspergillus oryzae* Lacking Both α-1,3-Glucan and Galactosaminogalactan in Batch Culture with a Lab-Scale Bioreactor. J. Biosci. Bioeng..

[9] Susukida S., Miyazawa K., Ichikawa H., Muto K., Yoshimi A., Kumagai T., Kato Y., Abe K. (2025). Improved Mixing Properties of Stirred Fermentation of an *Aspergillus oryzae* Hyphal Dispersion Mutant. Biotechnol. Bioeng..

[10] Lu H., Cao W., Liu X., Sui Y., Ouyang L., Xia J., Huang M., Zhuang Y., Zhang S., Noorman H. (2018). Multi-Omics Integrative Analysis with Genome-Scale Metabolic Model Simulation Reveals Global Cellular Adaptation of *Aspergillus niger* under Industrial Enzyme Production Condition. Sci. Rep..

[11] Masuo S., Terabayashi Y., Shimizu M., Fujii T., Kitazume T., Takaya N. (2010). Global Gene Expression Analysis of *Aspergillus nidulans* Reveals Metabolic Shift and Transcription Suppression under Hypoxia. Mol. Genet. Genom..

[12] Terabayashi Y., Shimizu M., Kitazume T., Masuo S., Fujii T., Takaya N. (2012). Conserved and Specific Responses to Hypoxia in *Aspergillus oryzae* and *Aspergillus nidulans* Determined by Comparative Transcriptomics. Appl. Microbiol. Biotechnol..

[13] Pedersen L., Hansen K., Nielsen J., Lantz A.E., Thykaer J. (2012). Industrial Glucoamylase Fed-batch Benefits from Oxygen Limitation and High Osmolarity. Biotechnol. Bioeng..

[14] Bendjilali N., MacLeon S., Kalra G., Willis S.D., Hossian A.K.M.N., Avery E., Wojtowicz O., Hickman M.J. (2017). Time-Course Analysis of Gene Expression during the Saccharomyces Cerevisiae Hypoxic Response. G3 Genes Genomes Genet..

[15] Losada L., Barker B.M., Pakala S., Pakala S., Joardar V., Zafar N., Mounaud S., Fedorova N., Nierman W.C., Cramer R.A. (2014). Large-Scale Transcriptional Response to Hypoxia in Aspergillus Fumigatus Observed Using RNAseq Identifies a Novel Hypoxia Regulated NcRNA. Mycopathologia.

[16] Barker B.M., Kroll K., Vödisch M., Mazurie A., Kniemeyer O., Cramer R.A. (2012). Transcriptomic and Proteomic Analyses of the *Aspergillus fumigatus* Hypoxia Response Using an Oxygen-Controlled Fermenter. BMC Genom..

[17] Hillmann F., Shekhova E., Kniemeyer O. (2015). Insights into the Cellular Responses to Hypoxia in Filamentous Fungi. Curr. Genet..

[18] Willger S.D., Puttikamonkul S., Kim K.-H., Burritt J.B., Grahl N., Metzler L.J., Barbuch R., Bard M., Lawrence C.B., Cramer R.A. (2008). A Sterol-Regulatory Element Binding Protein Is Required for Cell Polarity, Hypoxia Adaptation, Azole Drug Resistance, and Virulence in *Aspergillus fumigatus*. PLoS Pathog..

[19] Nolfi-Donegan D., Braganza A., Shiva S. (2020). Mitochondrial Electron Transport Chain: Oxidative Phosphorylation, Oxidant Production, and Methods of Measurement. Redox Biol..

[20] Murphy M.P. (2009). How Mitochondria Produce Reactive Oxygen Species. Biochem. J..

[21] Shekhova E., Ivanova L., Krüger T., Stroe M.C., Macheleidt J., Kniemeyer O., Brakhage A.A. (2019). Redox Proteomic Analysis Reveals Oxidative Modifications of Proteins by Increased Levels of Intracellular Reactive Oxygen Species during Hypoxia Adaptation of *Aspergillus fumigatus*. Proteomics.

[22] Flipphi M., Márton A., Bíró V., Ág N., Sándor E., Fekete E., Karaffa L. (2023). Generation, Transfer, and Loss of Alternative Oxidase Paralogues in the *Aspergillaceae* Family. J. Fungi.

[23] Li J., Yang S., Wu Y., Wang R., Liu Y., Liu J., Ye Z., Tang R., Whiteway M., Lv Q. (2024). Alternative Oxidase: From Molecule and Function to Future Inhibitors. ACS Omega.

[24] Miyazawa K., Yoshimi A., Zhang S., Sano M., Nakayama M., Gomi K., Abe K. (2016). Increased Enzyme Production under Liquid Culture Conditions in the Industrial Fungus *Aspergillus oryzae* by Disruption of the Genes Encoding Cell Wall α-1,3-Glucan Synthase. Biosci. Biotechnol. Biochem..

[25] Chen S., Zhou Y., Chen Y., Gu J. (2018). Fastp: An Ultra-Fast All-in-One FASTQ Preprocessor. Proceedings of the Bioinformatics.

[26] Dobin A., Davis C.A., Schlesinger F., Drenkow J., Zaleski C., Jha S., Batut P., Chaisson M., Gingeras T.R. (2013). STAR: Ultrafast Universal RNA-Seq Aligner. Bioinformatics.

[27] Liao Y., Smyth G.K., Shi W. (2014). FeatureCounts: An Efficient General Purpose Program for Assigning Sequence Reads to Genomic Features. Bioinformatics.

[28] Ashburner M., Ball C.A., Blake J.A., Botstein D., Butler H., Cherry J.M., Davis A.P., Dolinski K., Dwight S.S., Eppig J.T. (2000). Gene Ontology: Tool for the Unification of Biology. Nat. Genet..

[29] Aleksander S.A., Balhoff J., Carbon S., Cherry J.M., Drabkin H.J., Ebert D., Feuermann M., Gaudet P., Harris N.L., Hill D.P. (2023). The Gene Ontology Knowledgebase in 2023. Genetics.

[30] Schindelin J., Arganda-Carreras I., Frise E., Kaynig V., Longair M., Pietzsch T., Preibisch S., Rueden C., Saalfeld S., Schmid B. (2012). Fiji: An Open-Source Platform for Biological-Image Analysis. Nat. Methods.

[31] Otsu N. (1979). A Threshold Selection Method from Gray-Level Histograms. IEEE Trans. Syst. Man. Cybern..

[32] Stincone A., Prigione A., Cramer T., Wamelink M.M.C., Campbell K., Cheung E., Olin-Sandoval V., Grüning N.M., Krüger A., Tauqeer Alam M. (2015). The Return of Metabolism: Biochemistry and Physiology of the Pentose Phosphate Pathway. Biol. Rev..

[33] Shimizu M., Fujii T., Masuo S., Fujita K., Takaya N. (2009). Proteomic Analysis of *Aspergillus nidulans* Cultured under Hypoxic Conditions. Proteomics.

[34] Burgain A., Tebbji F., Khemiri I., Sellam A. (2020). Metabolic Reprogramming in the Opportunistic Yeast *Candida albicans* in Response to Hypoxia. mSphere.

[35] Rintala E., Toivari M., Pitkänen J.-P., Wiebe M.G., Ruohonen L., Penttilä M. (2009). Low Oxygen Levels as a Trigger for Enhancement of Respiratory Metabolism in *Saccharomyces cerevisiae*. BMC Genom..

[36] Wamelink M.M.C., Struys E.A., Huck J.H.J., Roos B., Van Der Knaap M.S., Jakobs C., Verhoeven N.M. (2005). Quantification of Sugar Phosphate Intermediates of the Pentose Phosphate Pathway by LC–MS/MS: Application to Two New Inherited Defects of Metabolism. J. Chromatogr. B.

[37] Morano K.A., Grant C.M., Moye-Rowley W.S. (2012). The Response to Heat Shock and Oxidative Stress in *Saccharomyces cerevisiae*. Genetics.

[38] Vödisch M., Scherlach K., Winkler R., Hertweck C., Braun H.P., Roth M., Haas H., Werner E.R., Brakhage A.A., Kniemeyer O. (2011). Analysis of the *Aspergillus fumigatus* Proteome Reveals Metabolic Changes and the Activation of the Pseurotin A Biosynthesis Gene Cluster in Response to Hypoxia. J. Proteome Res..

[39] Chung D., Barker B.M., Carey C.C., Merriman B., Werner E.R., Lechner B.E., Dhingra S., Cheng C., Xu W., Blosser S.J. (2014). ChIP-Seq and In Vivo Transcriptome Analyses of the *Aspergillus fumigatus* SREBP SrbA Reveals a New Regulator of the Fungal Hypoxia Response and Virulence. PLoS Pathog..

[40] Dietz J.V., Fox J.L., Khalimonchuk O. (2021). Down the Iron Path: Mitochondrial Iron Homeostasis and Beyond. Cells.

[41] Lill R., Freibert S.-A. (2020). Mechanisms of Mitochondrial Iron-Sulfur Protein Biogenesis. Annu. Rev. Biochem..

[42] Arias-Mayenco I., González-Rodríguez P., Torres-Torrelo H., Gao L., Fernández-Agüera M.C., Bonilla-Henao V., Ortega-Sáenz P., López-Barneo J. (2018). Acute O2 Sensing: Role of Coenzyme QH2/Q Ratio and Mitochondrial ROS Compartmentalization. Cell Metab..

[43] Chandel N.S., McClintock D.S., Feliciano C.E., Wood T.M., Melendez J.A., Rodriguez A.M., Schumacker P.T. (2000). Reactive Oxygen Species Generated at Mitochondrial Complex III Stabilize Hypoxia-Inducible Factor-1α during Hypoxia: A Mechanism of O2 Sensing. J. Biol. Chem..

[44] Guzy R.D., Hoyos B., Robin E., Chen H., Liu L., Mansfield K.D., Simon M.C., Hammerling U., Schumacker P.T. (2005). Mitochondrial Complex III Is Required for Hypoxia-Induced ROS Production and Cellular Oxygen Sensing. Cell Metab..

[45] Robb E.L., Hall A.R., Prime T.A., Eaton S., Szibor M., Viscomi C., James A.M., Murphy M.P. (2018). Control of Mitochondrial Superoxide Production by Reverse Electron Transport at Complex I. J. Biol. Chem..

[46] Neikirk K., Marshall A.G., Kula B., Smith N., LeBlanc S., Hinton A. (2023). MitoTracker: A Useful Tool in Need of Better Alternatives. Eur. J. Cell Biol..

[47] Joseph-Horne T., Hollomon D.W., Wood P.M. (2001). Fungal Respiration: A Fusion of Standard and Alternative Components. Biochim. Biophys. Acta Bioenerg..

[48] Tian F., Lee S.Y., Woo S.Y., Chun H.S. (2020). Alternative Oxidase: A Potential Target for Controlling Aflatoxin Contamination and Propagation of *Aspergillus flavus*. Front. Microbiol..

[49] Wood P.M., Hollomon D.W. (2003). A Critical Evaluation of the Role of Alternative Oxidase in the Performance of Strobilurin and Related Fungicides Acting at the Qo Site of Complex III. Pest. Manag. Sci..

[50] Grahl N., Dinamarco T.M., Willger S.D., Goldman G.H., Cramer R.A. (2012). *Aspergillus fumigatus* Mitochondrial Electron Transport Chain Mediates Oxidative Stress Homeostasis, Hypoxia Responses and Fungal Pathogenesis. Mol. Microbiol..

[51] Purvis A.C. (1997). Role of the Alternative Oxidase in Limiting Superoxide Production by Plant Mitochondria. Physiol. Plant..

[52] Wagner A.M., Moore A.L. (1997). Structure and Function of the Plant Alternative Oxidase: Its Putative Role in the Oxygen Defence Mechanism. Biosci. Rep..

[53] Leiter É., Park H.-S., Kwon N.-J., Han K.-H., Emri T., Oláh V., Mészáros I., Dienes B., Vincze J., Csernoch L. (2016). Characterization of the AodA, DnmA, MnSOD and PimA Genes in *Aspergillus nidulans*. Sci. Rep..

[54] El-Khoury R., Dufour E., Rak M., Ramanantsoa N., Grandchamp N., Csaba Z., Duvillié B., Bénit P., Gallego J., Gressens P. (2013). Alternative Oxidase Expression in the Mouse Enables Bypassing Cytochrome c Oxidase Blockade and Limits Mitochondrial ROS Overproduction. PLoS Genet..

[55] Magnani T., Soriani F.M., Martins V.D.P., Policarpo A.C.D.F., Sorgi C.A., Faccioli L.H., Curti C., Uyemura S.A. (2008). Silencing of Mitochondrial Alternative Oxidase Gene of *Aspergillus fumigatus* Enhances Reactive Oxygen Species Production and Killing of the Fungus by Macrophages. J. Bioenerg. Biomembr..

[56] Maxwell D.P., Wang Y., McIntosh L. (1999). The Alternative Oxidase Lowers Mitochondrial Reactive Oxygen Production in Plant Cells. Proc. Natl. Acad. Sci. USA.

[57] Carmel-Harel O., Storz G. (2000). Roles of the Glutathione- and Thioredoxin-Dependent Reduction Systems in the *Escherichia coli* and *Saccharomyces cerevisiae* Responses to Oxidative Stress. Annu. Rev. Microbiol..

[58] Trotter E.W., Grant C.M. (2003). Non-Reciprocal Regulation of the Redox State of the Glutathione-Glutaredoxin and Thioredoxin Systems. EMBO Rep..

[59] Couto N., Wood J., Barber J. (2016). The Role of Glutathione Reductase and Related Enzymes on Cellular Redox Homoeostasis Network. Free Radic. Biol. Med..

[60] Kwolek-Mirek M., Maslanka R., Bednarska S., Przywara M., Kwolek K., Zadrag-Tecza R. (2024). Strategies to Maintain Redox Homeostasis in Yeast Cells with Impaired Fermentation-Dependent NADPH Generation. Int. J. Mol. Sci..

[61] Kwast K.E., Lai L.C., Menda N., James D.T., Aref S., Burke P.V. (2002). Genomic Analyses of Anaerobically Induced Genes in *Saccharomyces cerevisiae*: Functional Roles of Rox1 and Other Factors in Mediating the Anoxic Response. J. Bacteriol..

[62] Flood D., Lee E.S., Taylor C.T. (2023). Intracellular Energy Production and Distribution in Hypoxia. J. Biol. Chem..

