# Supplementary material for: Analysis of Short-Term Responses to Hypoxia During Stirred-Tank Fermentation in Aspergillus oryzae
Source: J Fungi (Basel). 2026 May 7;12(5):347. doi: 10.3390/jof12050347 (PMC13208471; doi:10.3390/jof12050347)
Supplement: Supplementary file 1 [file jof-12-00347-s001.zip › Supplementary Figures.pdf]

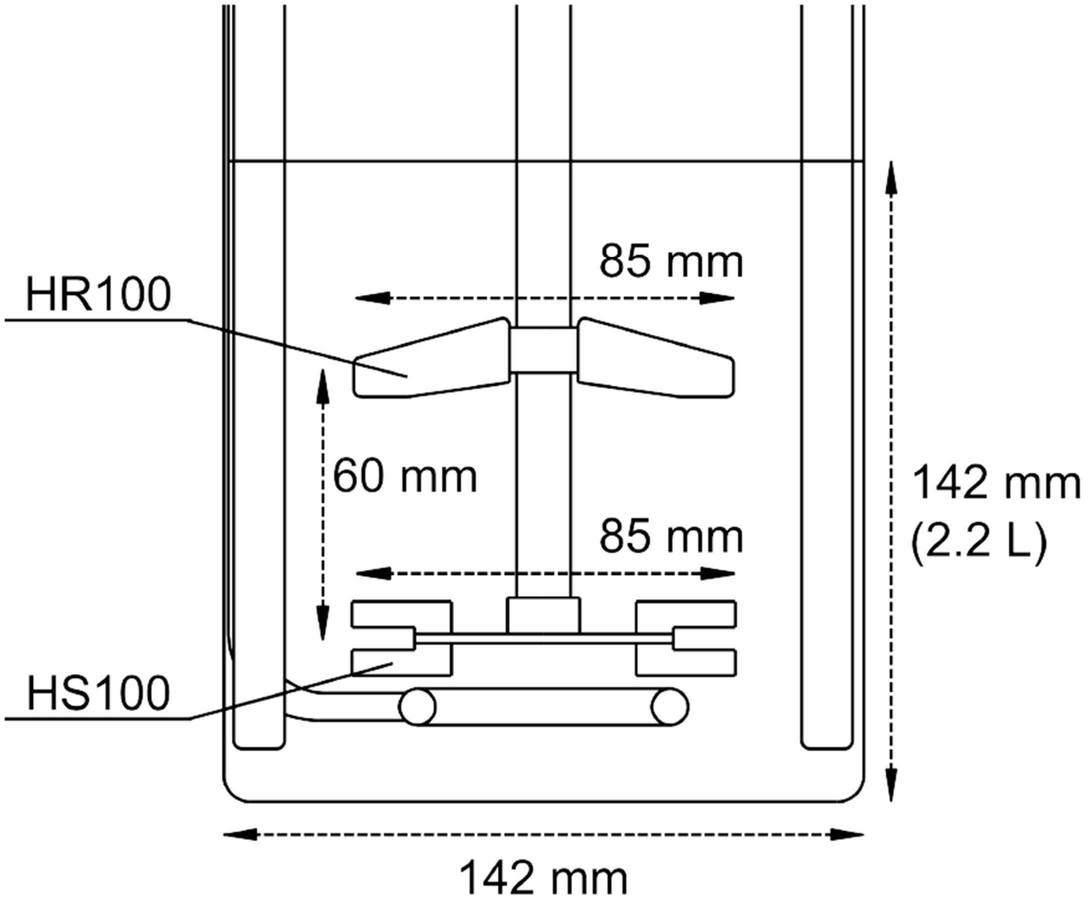

Figure S1. Schematic diagram of the 4 L bioreactor.

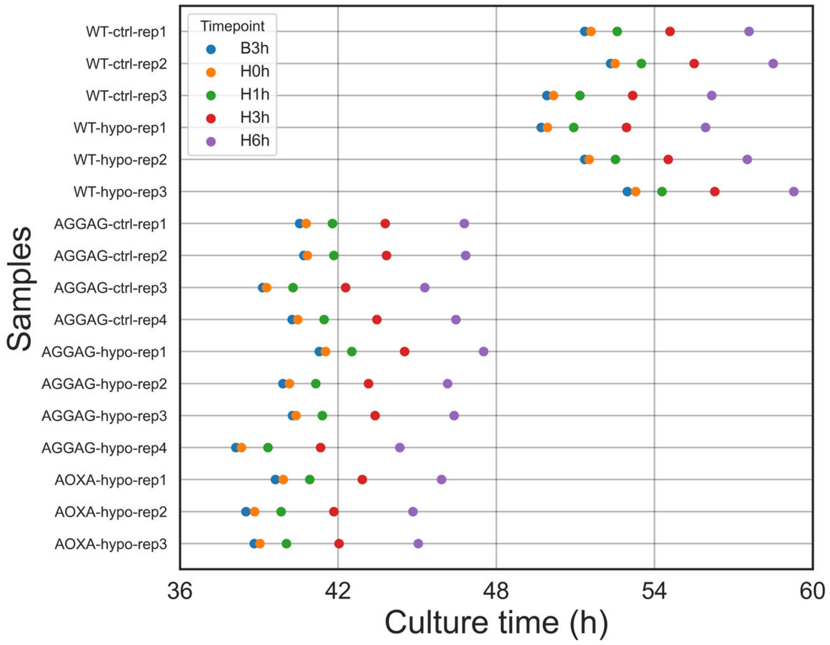

Fig. S2. Actual sampling timepoints of the WT, AGΔ-GAGΔ, and AGΔ-GAGΔ-ΔAOXA cultures. AGGAG, AGΔ-GAGΔ; AOXA, AGΔ-GAGΔ-ΔAOXA; hypo, hypoxic culture; ctrl, control culture.

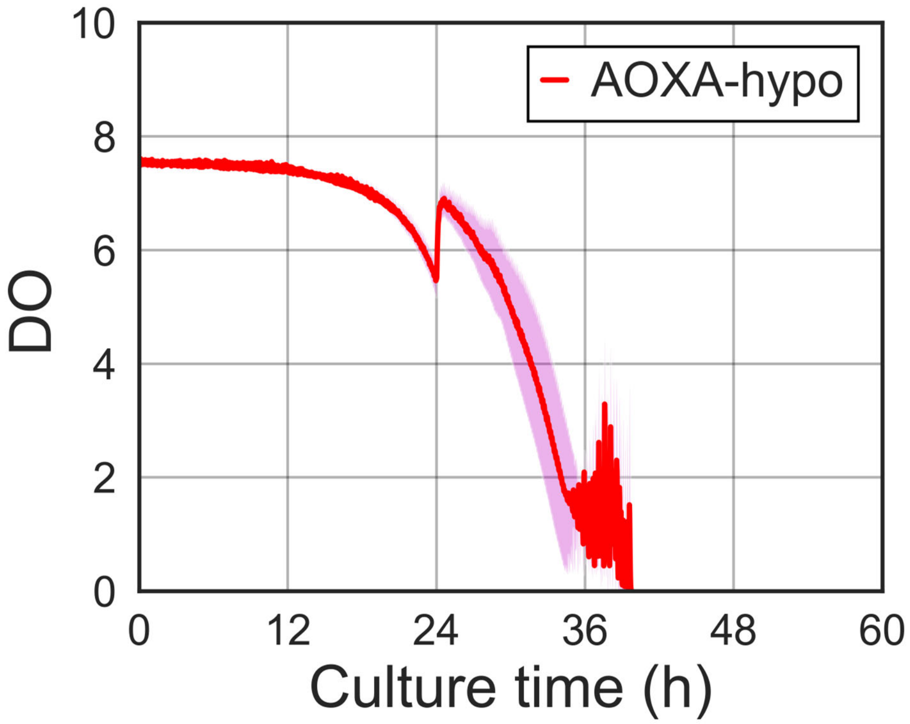

AOXA-hypo
DO
Culture time (h)

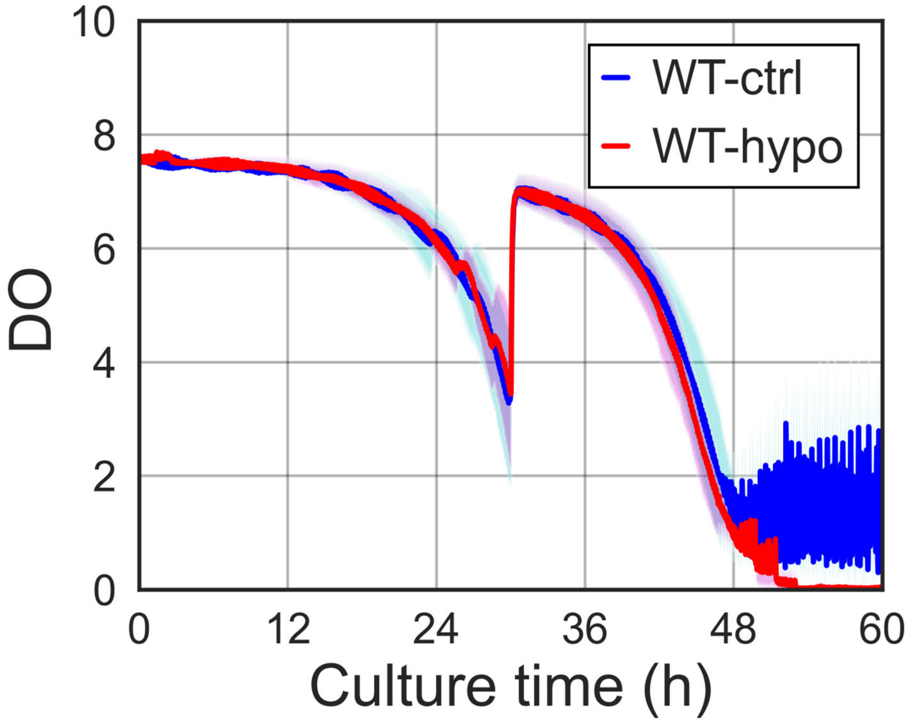

WT-ctrl
WT-hypo
DO
Culture time (h)

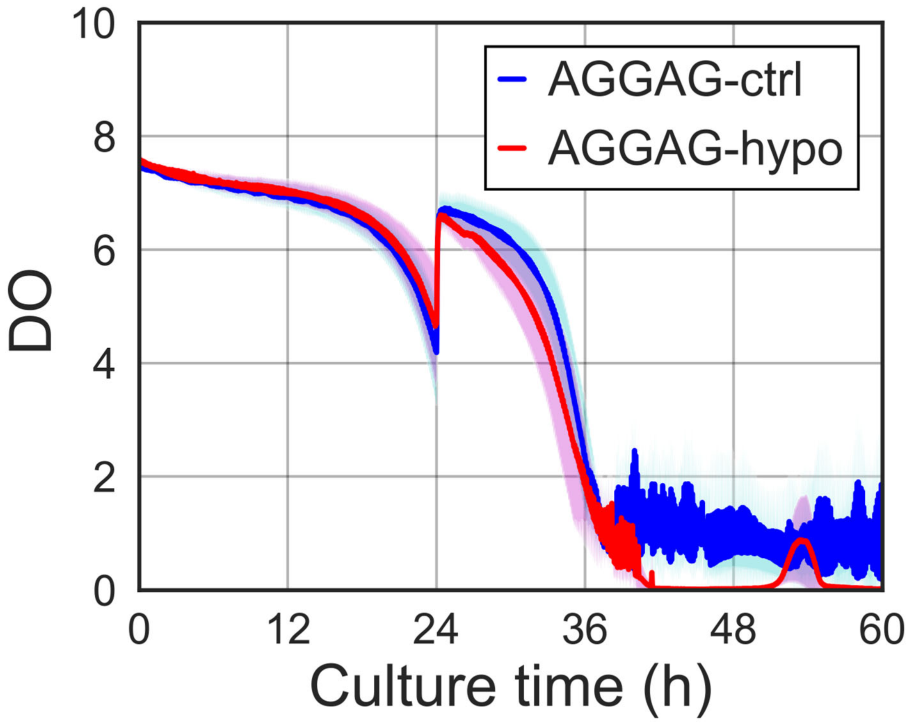

Fig. S3. Dissolved oxygen (DO) traces of the WT, AGΔ-GAGΔ, and AGΔ-GAGΔ-ΔAOXA cultures. Solid lines, mean values of three biological replicates; shading, standard deviation. AGGAG, AGΔ-GAGΔ; AOXA, AGΔ-GAGΔ-ΔAOXA; hypo, hypoxic culture; ctrl, control culture.

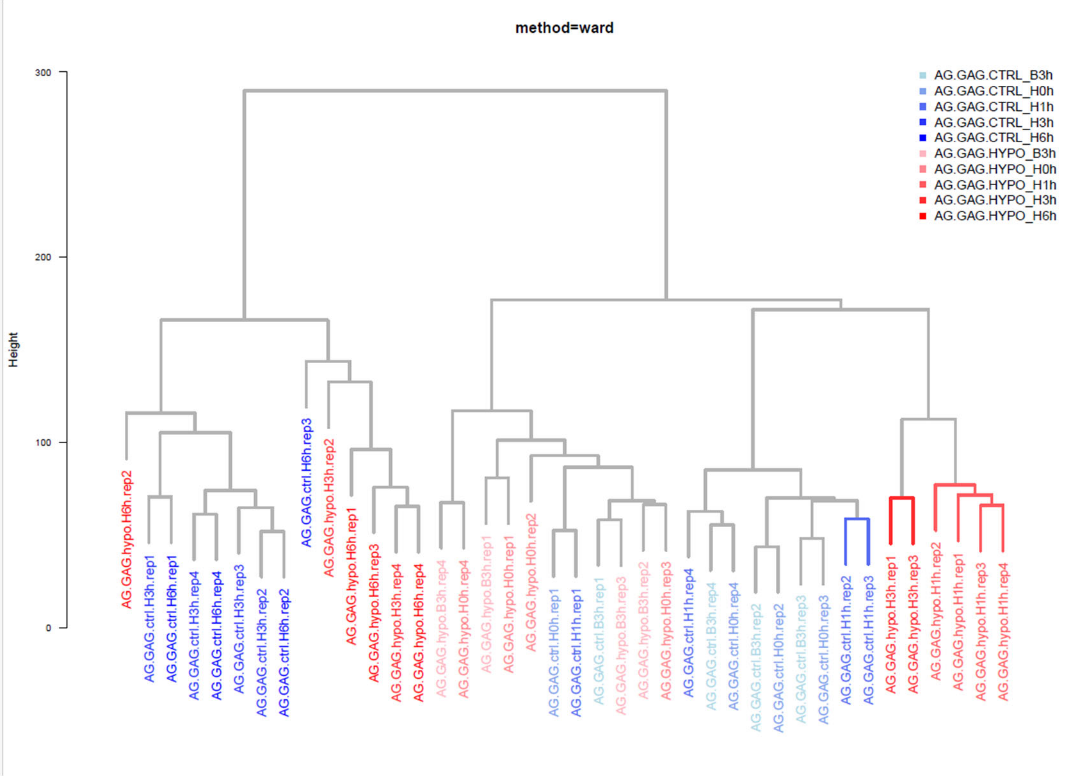

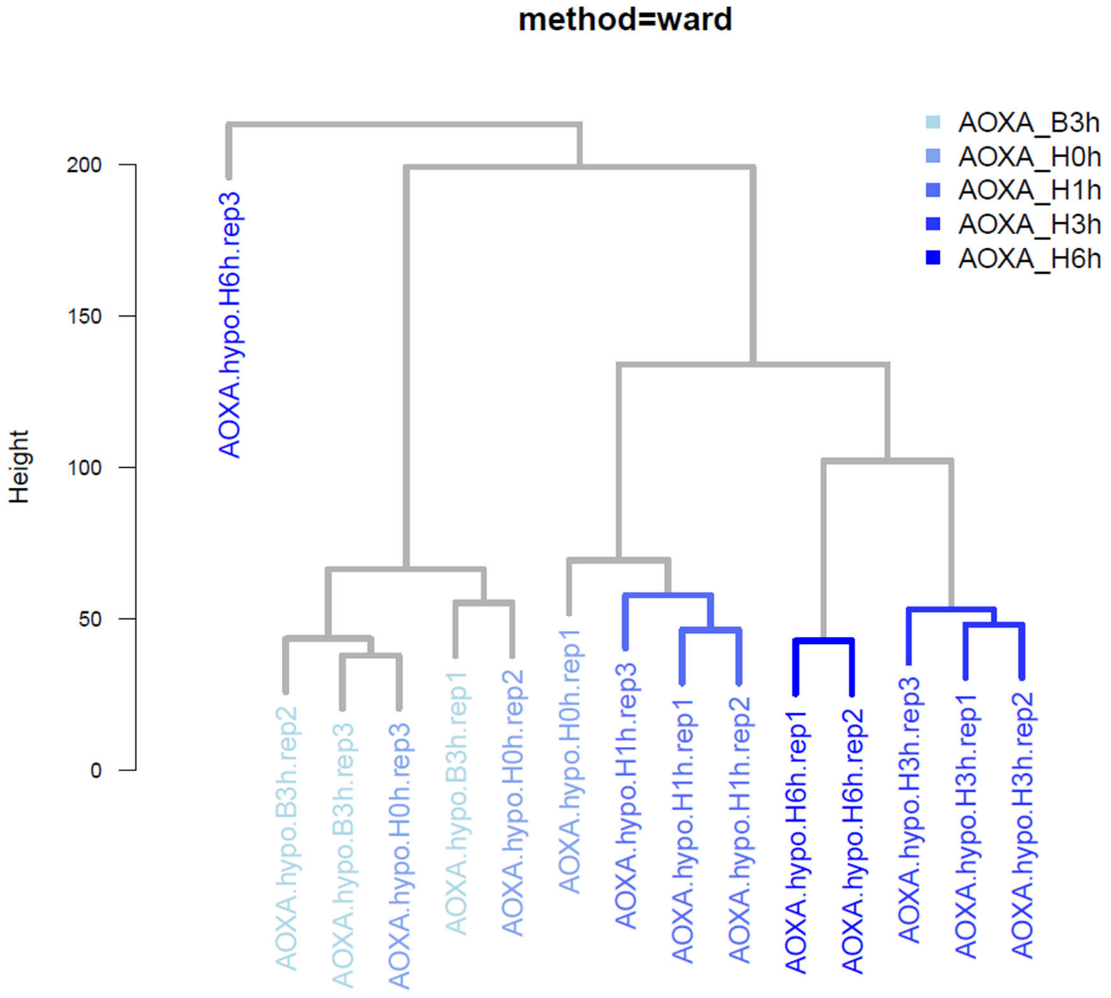
method=ward
Height
200
150
100
50
0
AOXA_B3h
AOXA_H0h
AOXA_H1h
AOXA_H3h
AOXA_H6h
AOXA.hypo.H6h.rep3
AOXA.hypo.B3h.rep2
AOXA.hypo.B3h.rep3
AOXA.hypo.H0h.rep3
AOXA.hypo.B3h.rep1
AOXA.hypo.H0h.rep2
AOXA.hypo.H0h.rep1
AOXA.hypo.H1h.rep3
AOXA.hypo.H1h.rep1
AOXA.hypo.H1h.rep2
AOXA.hypo.H6h.rep1
AOXA.hypo.H6h.rep2
AOXA.hypo.H3h.rep3
AOXA.hypo.H3h.rep1
AOXA.hypo.H3h.rep2

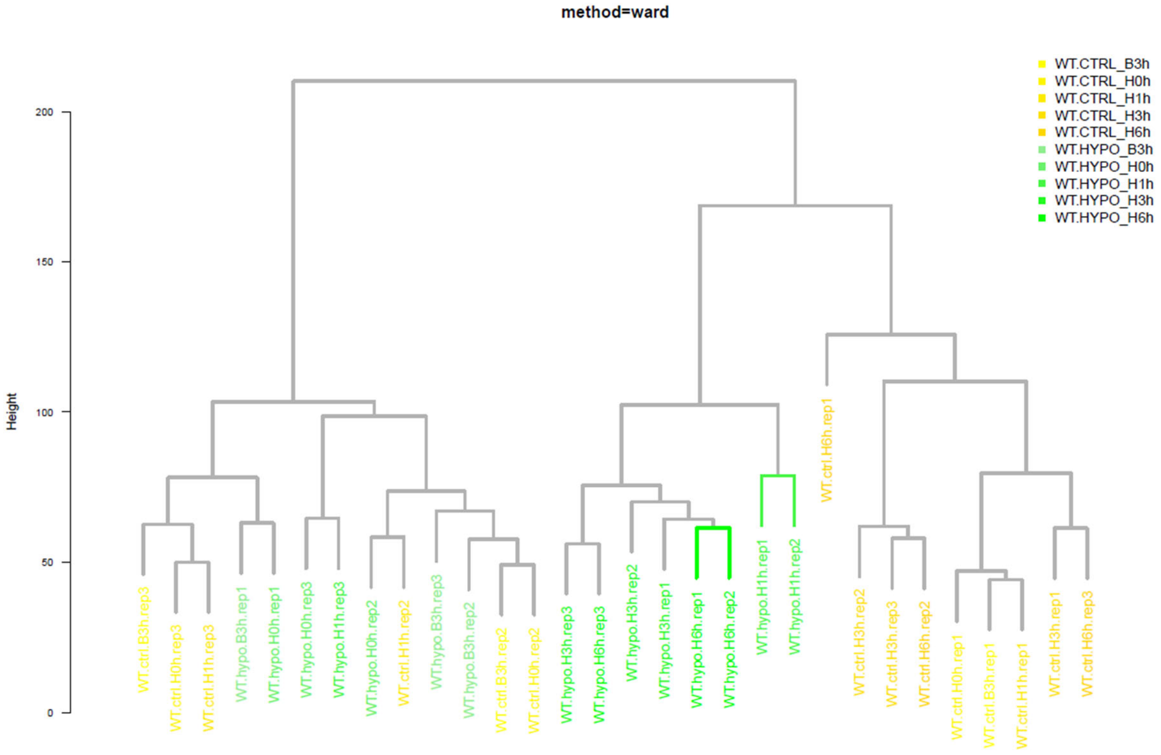
method=ward
Height
200
150
100
50
0
WT.CTRL_B3h
WT.CTRL_H0h
WT.CTRL_H1h
WT.CTRL_H3h
WT.CTRL_H6h
WT.HYPO_B3h
WT.HYPO_H0h
WT.HYPO_H1h
WT.HYPO_H3h
WT.HYPO_H6h

Fig. S4. Hierarchical clustering of RNA-seq samples across different timepoints. The dendrograms were constructed using the Ward linkage method based on the Euclidean distances between log2CPM values of genes of (A) WT, (B) AGΔ-GAGΔ, (C) AGΔ-GAGΔ-ΔAOXA. The *y*-axes represent the Euclidean distance. HYPO, hypoxic culture; CTRL, control culture.

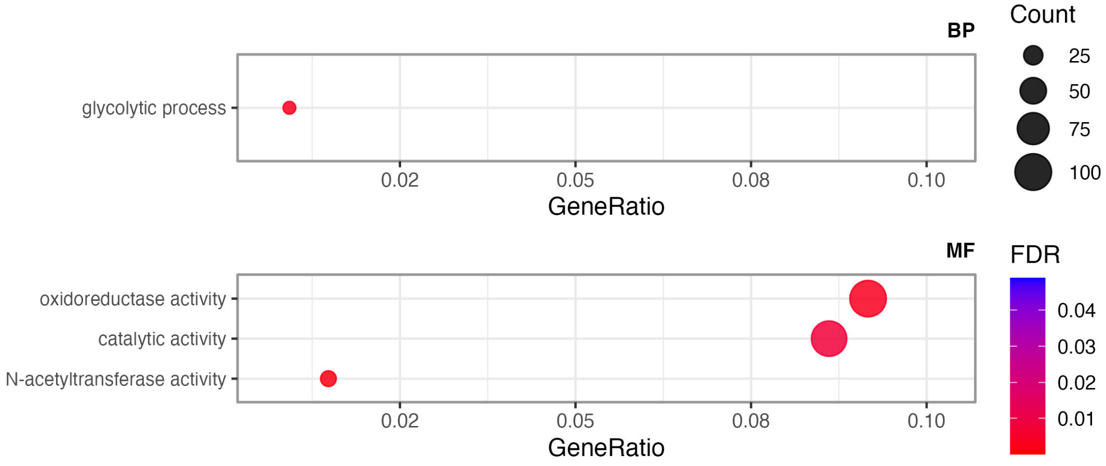

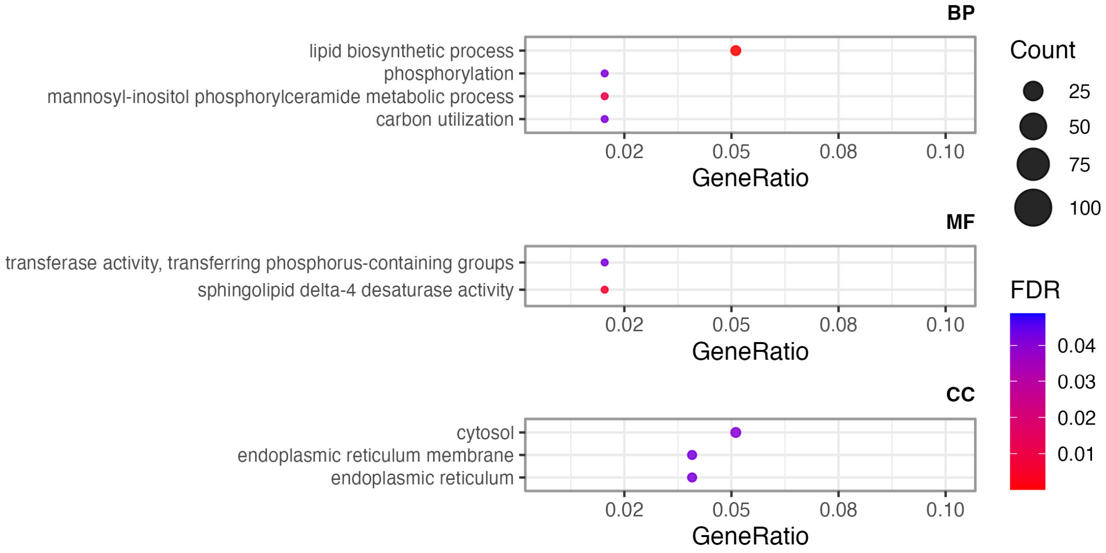

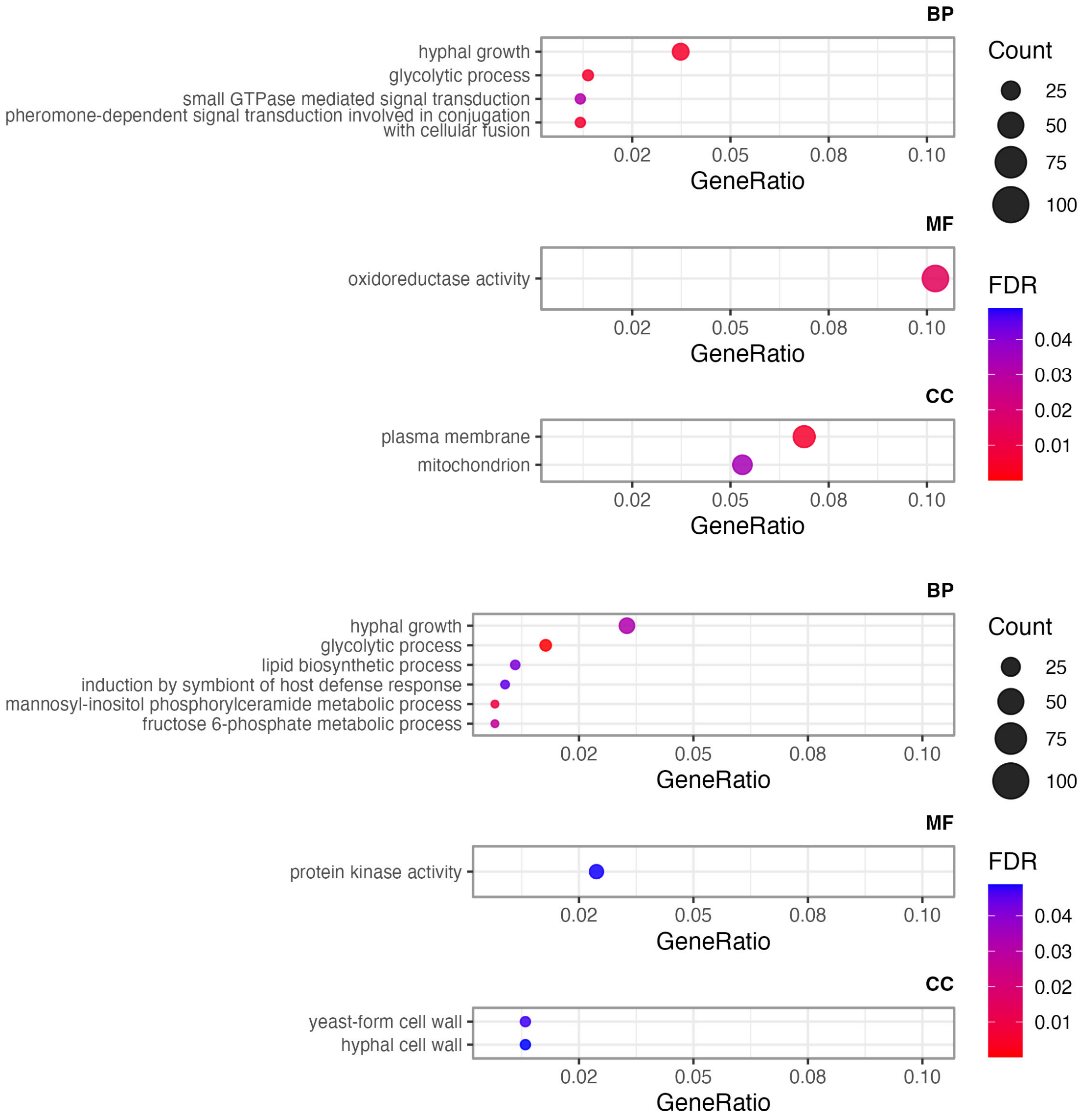

Fig. S5. Gene Ontology (GO) term enrichment across timepoints in WT. Comparisons: hypoxia vs. control. GO terms with false discovery rate (FDR) < 0.05 are shown; no GO terms were enriched at B3h. BP, biological process; MF, molecular function; CC, cellular component.

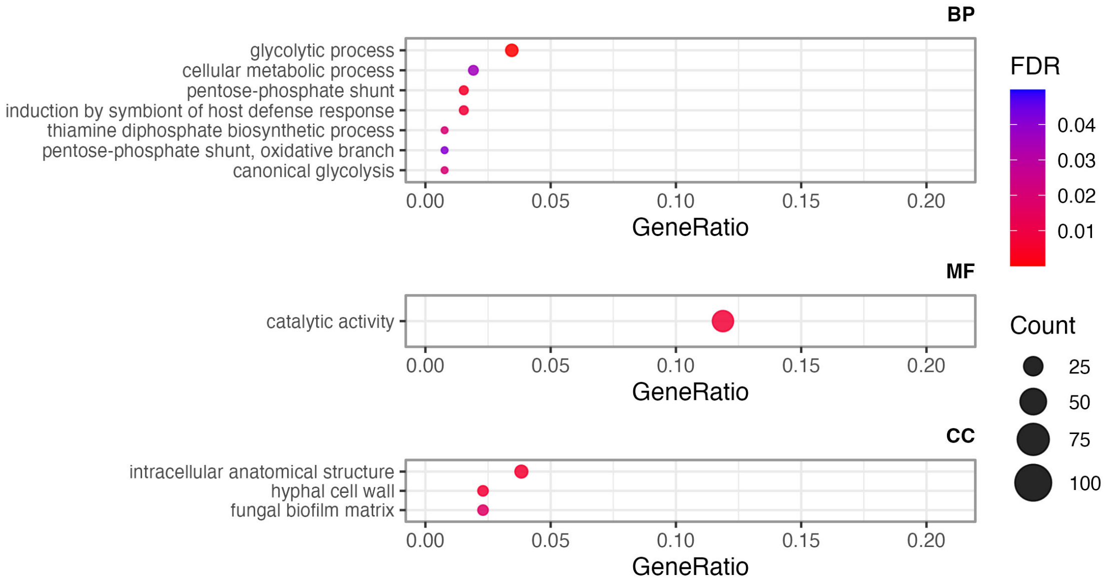
BP
glycolytic process
cellular metabolic process
pentose-phosphate shunt
induction by symbiont of host defense response
thiamine diphosphate biosynthetic process
pentose-phosphate shunt, oxidative branch
canonical glycolysis
0.00
0.05
0.10
0.15
0.20
GeneRatio
MF
catalytic activity
CC
intracellular anatomical structure
hyphal cell wall
fungal biofilm matrix
FDR
0.04
0.03
0.02
0.01
Count
25
50
75
100

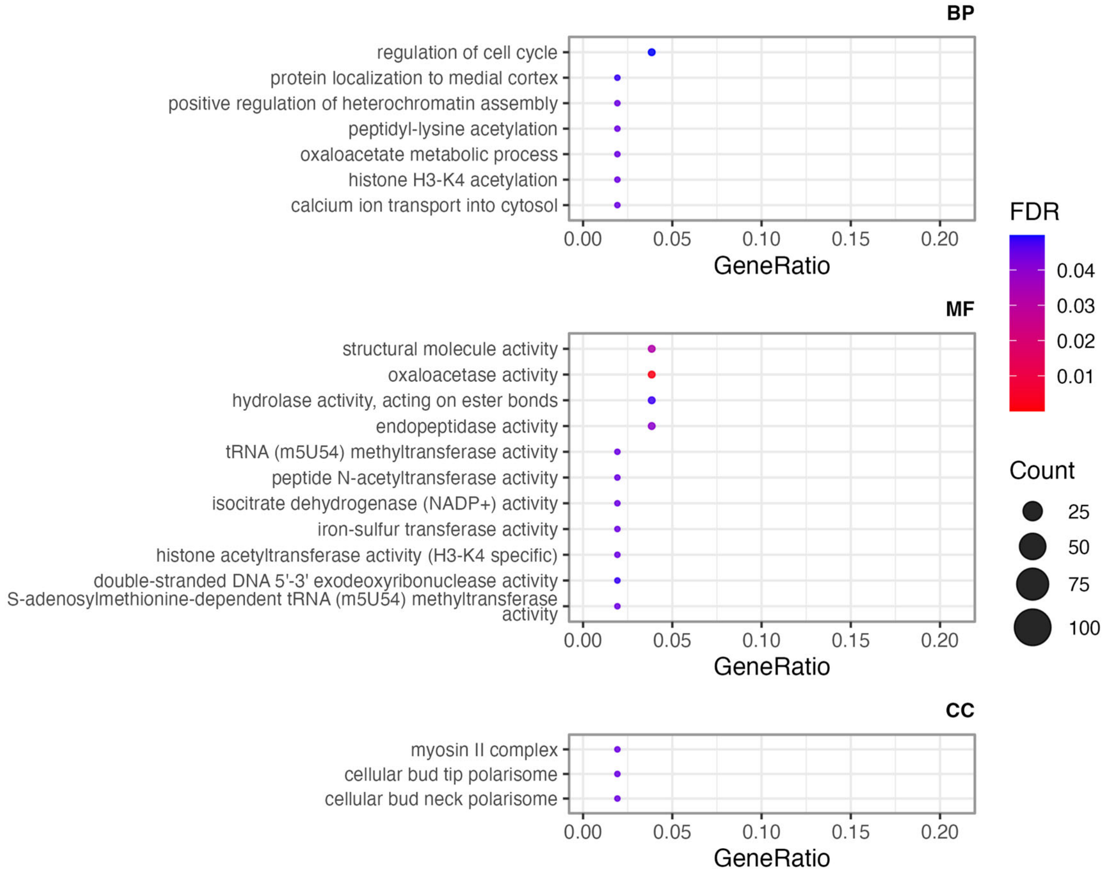
BP
regulation of cell cycle
protein localization to medial cortex
positive regulation of heterochromatin assembly
peptidyl-lysine acetylation
oxaloacetate metabolic process
histone H3-K4 acetylation
calcium ion transport into cytosol
0.00
0.05
0.10
0.15
0.20
GeneRatio
MF
structural molecule activity
oxaloacetase activity
hydrolase activity, acting on ester bonds
endopeptidase activity
tRNA (m5U54) methyltransferase activity
peptide N-acetyltransferase activity
isocitrate dehydrogenase (NADP+) activity
iron-sulfur transferase activity
histone acetyltransferase activity (H3-K4 specific)
double-stranded DNA 5'-3' exodeoxyribonuclease activity
S-adenosylmethionine-dependent tRNA (m5U54) methyltransferase activity
CC
myosin II complex
cellular bud tip polarisome
cellular bud neck polarisome
FDR
0.04
0.03
0.02
0.01
Count
25
50
75
100

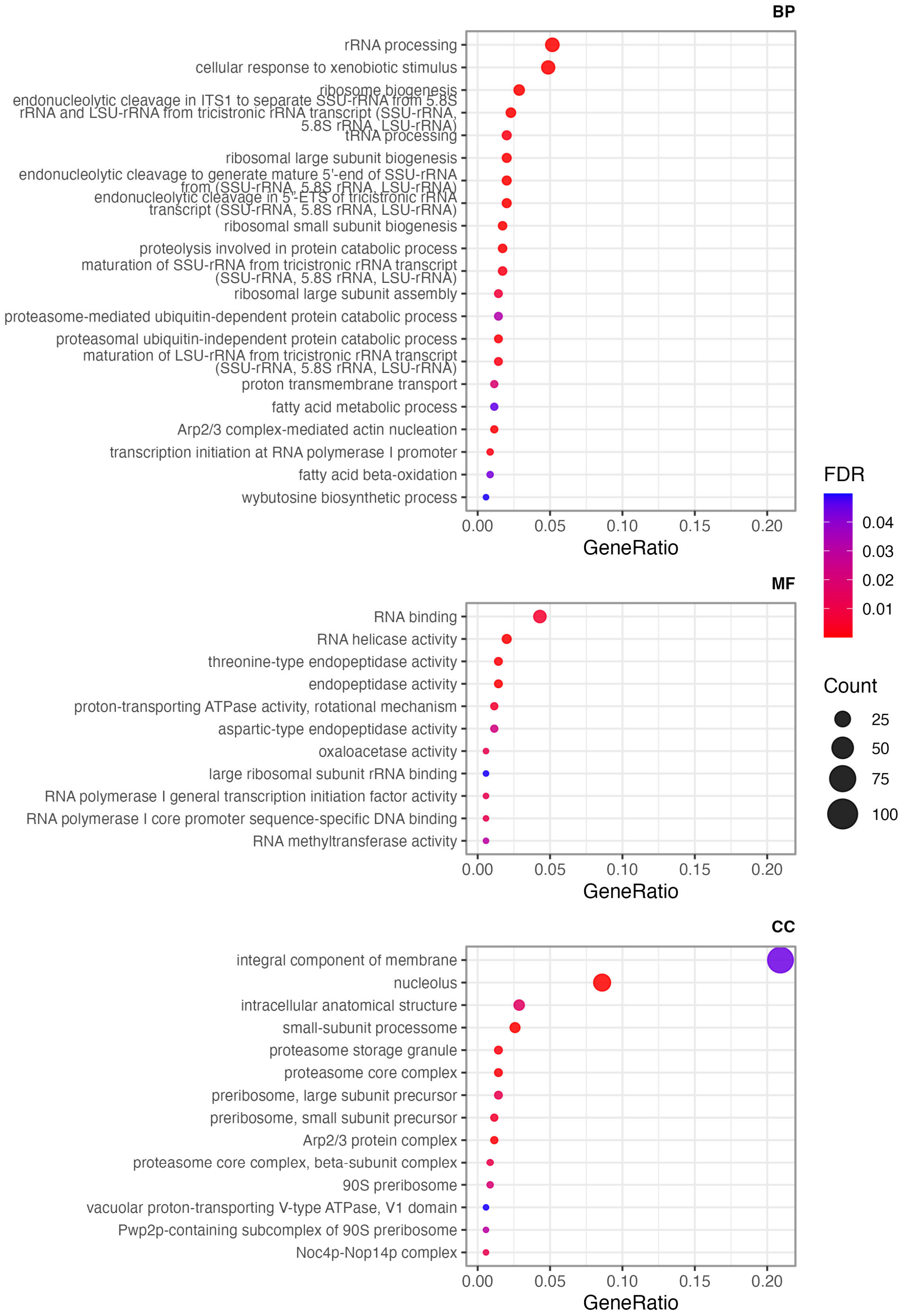
BP
rRNA processing
cellular response to xenobiotic stimulus
ribosome biogenesis
endonucleolytic cleavage in ITS1 to separate SSU-rRNA from 5.8S rRNA and LSU-rRNA from tricistronic rRNA transcript (SSU-rRNA, 5.8S rRNA, LSU-rRNA)
tRNA processing
ribosomal large subunit biogenesis
endonucleolytic cleavage to generate mature 5'-end of SSU-rRNA from (SSU-rRNA, 5.8S rRNA, LSU-rRNA)
endonucleolytic cleavage in 5'-ETS of tricistronic rRNA transcript (SSU-rRNA, 5.8S rRNA, LSU-rRNA)
ribosomal small subunit biogenesis
proteolysis involved in protein catabolic process
maturation of SSU-rRNA from tricistronic rRNA transcript (SSU-rRNA, 5.8S rRNA, LSU-rRNA)
ribosomal large subunit assembly
proteasome-mediated ubiquitin-dependent protein catabolic process
proteasomal ubiquitin-independent protein catabolic process
maturation of LSU-rRNA from tricistronic rRNA transcript (SSU-rRNA, 5.8S rRNA, LSU-rRNA)
proton transmembrane transport
fatty acid metabolic process
Arp2/3 complex-mediated actin nucleation
transcription initiation at RNA polymerase I promoter
fatty acid beta-oxidation
wybutosine biosynthetic process
0.00 0.05 0.10 0.15 0.20
GeneRatio
MF
RNA binding
RNA helicase activity
threonine-type endopeptidase activity
endopeptidase activity
proton-transporting ATPase activity, rotational mechanism
aspartic-type endopeptidase activity
oxaloacetase activity
large ribosomal subunit rRNA binding
RNA polymerase I general transcription initiation factor activity
RNA polymerase I core promoter sequence-specific DNA binding
RNA methyltransferase activity
0.00 0.05 0.10 0.15 0.20
GeneRatio
CC
integral component of membrane
nucleolus
intracellular anatomical structure
small-subunit processome
proteasome storage granule
proteasome core complex
preribosome, large subunit precursor
preribosome, small subunit precursor
Arp2/3 protein complex
proteasome core complex, beta-subunit complex
90S preribosome
vacuolar proton-transporting V-type ATPase, V1 domain
Pwp2p-containing subcomplex of 90S preribosome
Noc4p-Nop14p complex
0.00 0.05 0.10 0.15 0.20
GeneRatio
FDR
0.04
0.03
0.02
0.01
Count
25
50
75
100

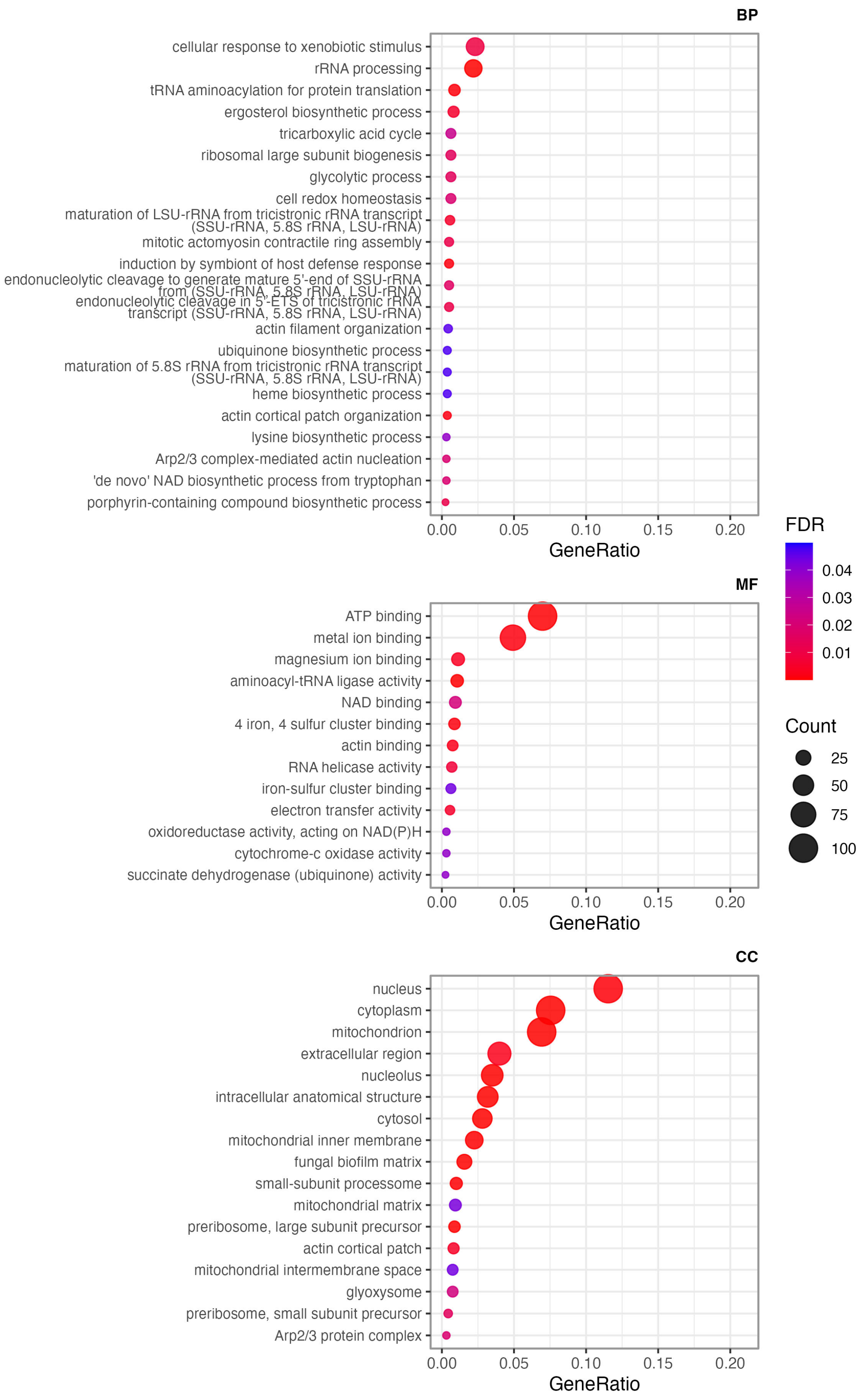
BP
cellular response to xenobiotic stimulus
rRNA processing
tRNA aminoacylation for protein translation
ergosterol biosynthetic process
tricarboxylic acid cycle
ribosomal large subunit biogenesis
glycolytic process
cell redox homeostasis
maturation of LSU-rRNA from tricistronic rRNA transcript (SSU-rRNA, 5.8S rRNA, LSU-rRNA)
mitotic actomyosin contractile ring assembly
induction by symbiont of host defense response
endonucleolytic cleavage to generate mature 5'-end of SSU-rRNA from (SSU-rRNA, 5.8S rRNA, LSU-rRNA)
endonucleolytic cleavage in 5'-ETS of tricistronic rRNA transcript (SSU-rRNA, 5.8S rRNA, LSU-rRNA)
actin filament organization
ubiquinone biosynthetic process
maturation of 5.8S rRNA from tricistronic rRNA transcript (SSU-rRNA, 5.8S rRNA, LSU-rRNA)
heme biosynthetic process
actin cortical patch organization
lysine biosynthetic process
Arp2/3 complex-mediated actin nucleation
'de novo' NAD biosynthetic process from tryptophan
porphyrin-containing compound biosynthetic process
0.00 0.05 0.10 0.15 0.20
GeneRatio
MF
ATP binding
metal ion binding
magnesium ion binding
aminoacyl-tRNA ligase activity
NAD binding
4 iron, 4 sulfur cluster binding
actin binding
RNA helicase activity
iron-sulfur cluster binding
electron transfer activity
oxidoreductase activity, acting on NAD(P)H
cytochrome-c oxidase activity
succinate dehydrogenase (ubiquinone) activity
0.00 0.05 0.10 0.15 0.20
GeneRatio
CC
nucleus
cytoplasm
mitochondrion
extracellular region
nucleolus
intracellular anatomical structure
cytosol
mitochondrial inner membrane
fungal biofilm matrix
small-subunit processome
mitochondrial matrix
preribosome, large subunit precursor
actin cortical patch
mitochondrial intermembrane space
glyoxysome
preribosome, small subunit precursor
Arp2/3 protein complex
0.00 0.05 0.10 0.15 0.20
GeneRatio
FDR
0.04
0.03
0.02
0.01
Count
25
50
75
100

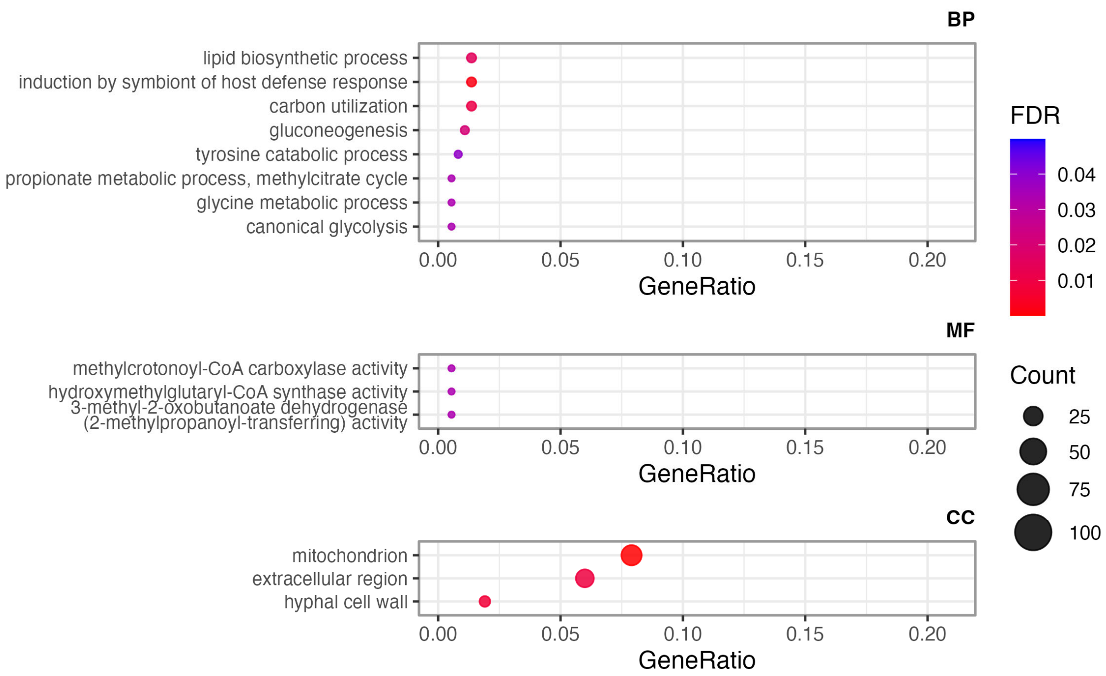

Fig. S6. Gene Ontology (GO) enrichment across timepoints in AGΔ-GAGΔ. Comparisons: hypoxia vs. control. GO terms with false discovery rate (FDR) < 0.05 are shown. BP, biological process; MF, molecular function; CC, cellular component.

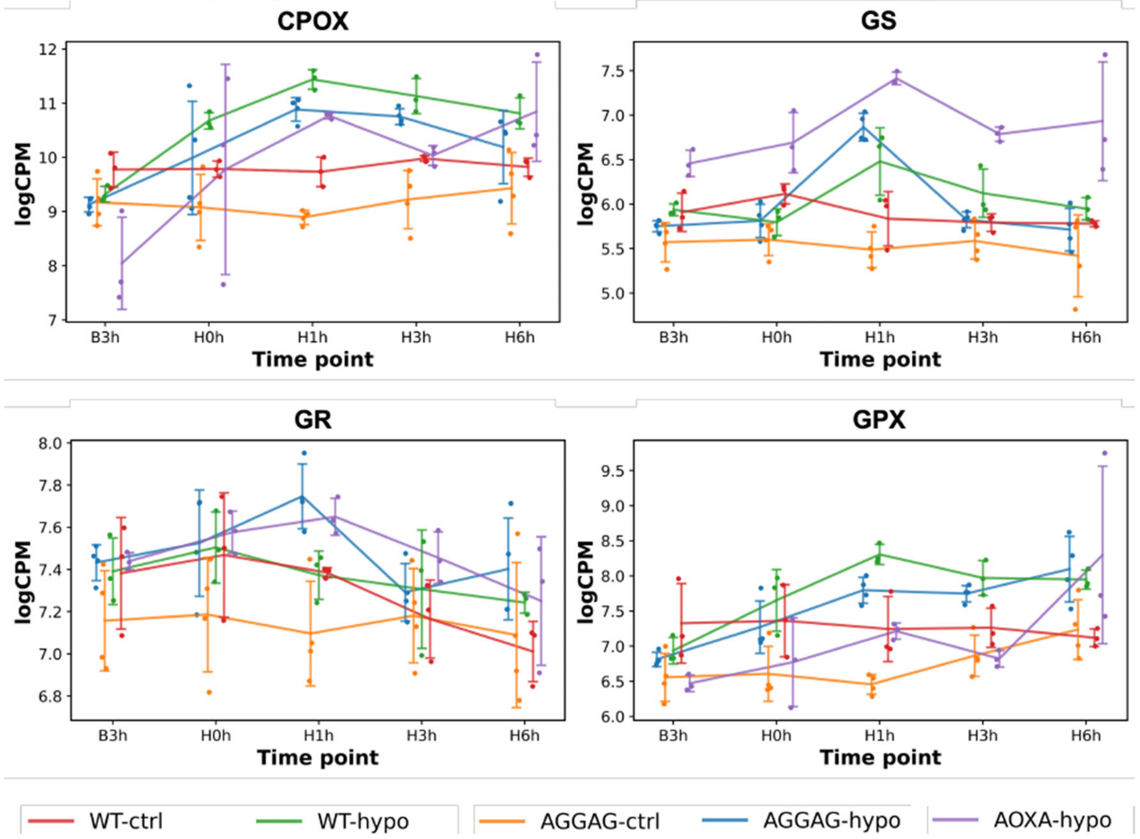

Fig. S7. Expression patterns of selected genes across timepoints in each strain and DO condition. CPOX, coproporphyrinogen III oxidase; GS, glutathione synthetase; GR, glutathione reductase; GPX, glutathione peroxidase. AGGAG, AGΔ-GAGΔ; AOXA, AGΔ-GAGΔ-ΔAOXA; hypo, hypoxic culture; ctrl, control culture.

A

Component		All genes	AGΔ-GAG ctrl		AGΔ-GAGΔ-ΔAOXA hypo		WT hypo		WT ctrl	
			DEGs	Ratio	DEGs	Ratio	DEGs	Ratio	DEGs	Ratio
	CI	23	0	0	1	0.043	0	0	0	0
	CII	4	0	0	2	0.5	0	0	0	0
	CIII	8	0	0	0	0	0	0	0	0
	Cyt c	1	0	0	0	0	0	0	0	0
	CIV	7	0	0	6	0.857	4	0.571	3	0.429
	CV	29	0	0	1	0.034	1	0.034	1	0.034

B

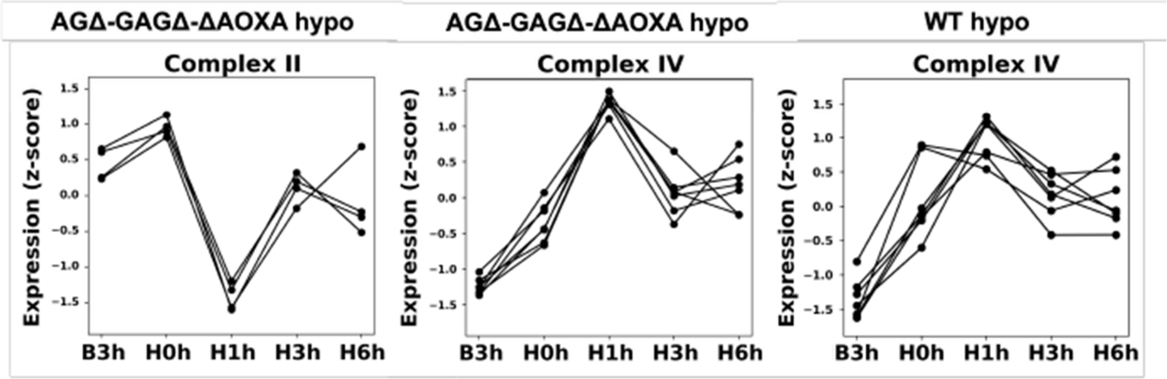

Fig. S8. Expression of mitochondrial respiratory chain genes in AGΔ-GAGΔ control culture, AGΔ-GAGΔ-ΔAOXA hypoxic culture, and in WT hypoxic and control cultures. (A) Numbers of differentially expressed genes (DEGs) and their ratios among all annotated genes for each. Components whose ratios of DEGs were ≥0.5 are shown in red. CI, Complex I; CII, Complex II; CIII, Complex III; Cyt c, Cytochrome *c*; CIV, Complex IV; CV, Complex V. (B) Z-score–standardized gene expression patterns of the respiratory components whose ratios of DEGs were ≥0.5. Hypo, hypoxic culture; ctrl, control culture.

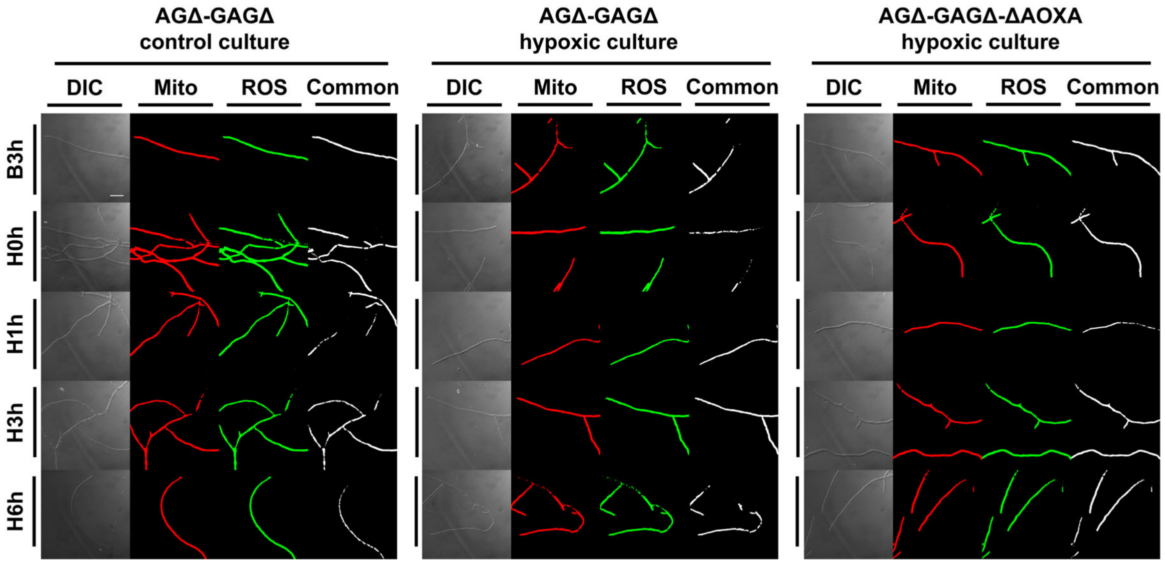

Fig. S9. Pixels selected by Otsu's method. DIC, differential interference contrast; Mito, filtered pixels for MitoTracker Deep Red fluorescence; ROS, filtered pixels for dichlorofluorescein (DCF) fluorescence; Common, pixels filtered by applying a common threshold obtained by Otsu's method to each sample. The same fields of view as in Figure 5 are shown. The Pearson correlation coefficient between the DCF and MitoTracker Deep Red fluorescence was calculated from the raw intensities of common pixels across all samples, and was 0.992. Scale bar = 20 µm.

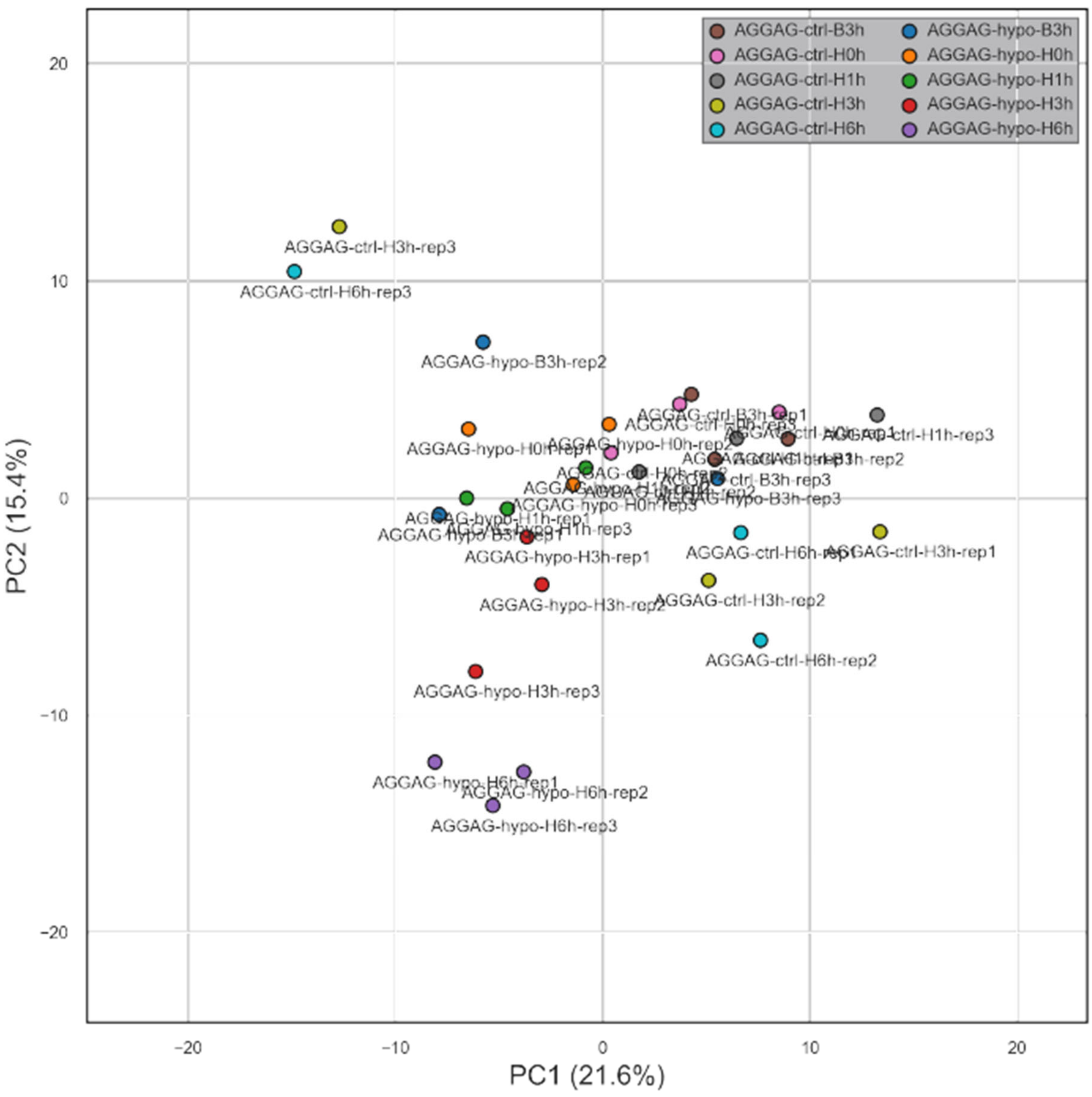

Fig. S10. Principal component analysis of metabolite profiles. Each point represents an individual sample, colored by DO condition and timepoint. PC1 and PC2, the first and second principal components; percentage values in parentheses indicate the proportion of variance explained by each component. AGGAG, AGΔ-GAGΔ; hypo, hypoxic culture; ctrl, control culture.

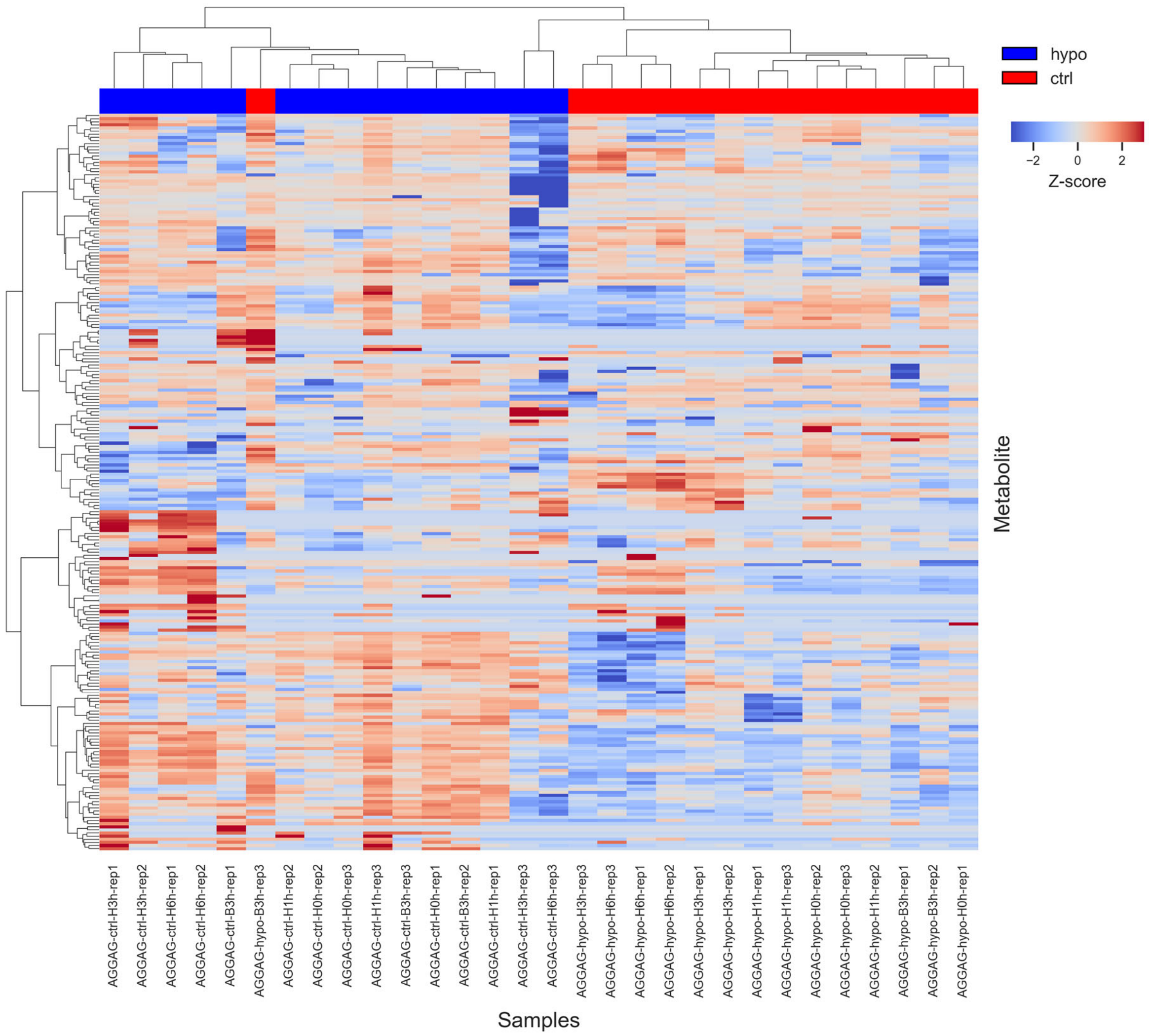

Fig. S11. Hierarchical clustering of metabolite profiles. Each row represents a metabolite. Color intensity reflects relative metabolite abundance based on Z-score normalized values. AGGAG, AGΔ-GAGΔ; hypo, hypoxic culture; ctrl, control culture.

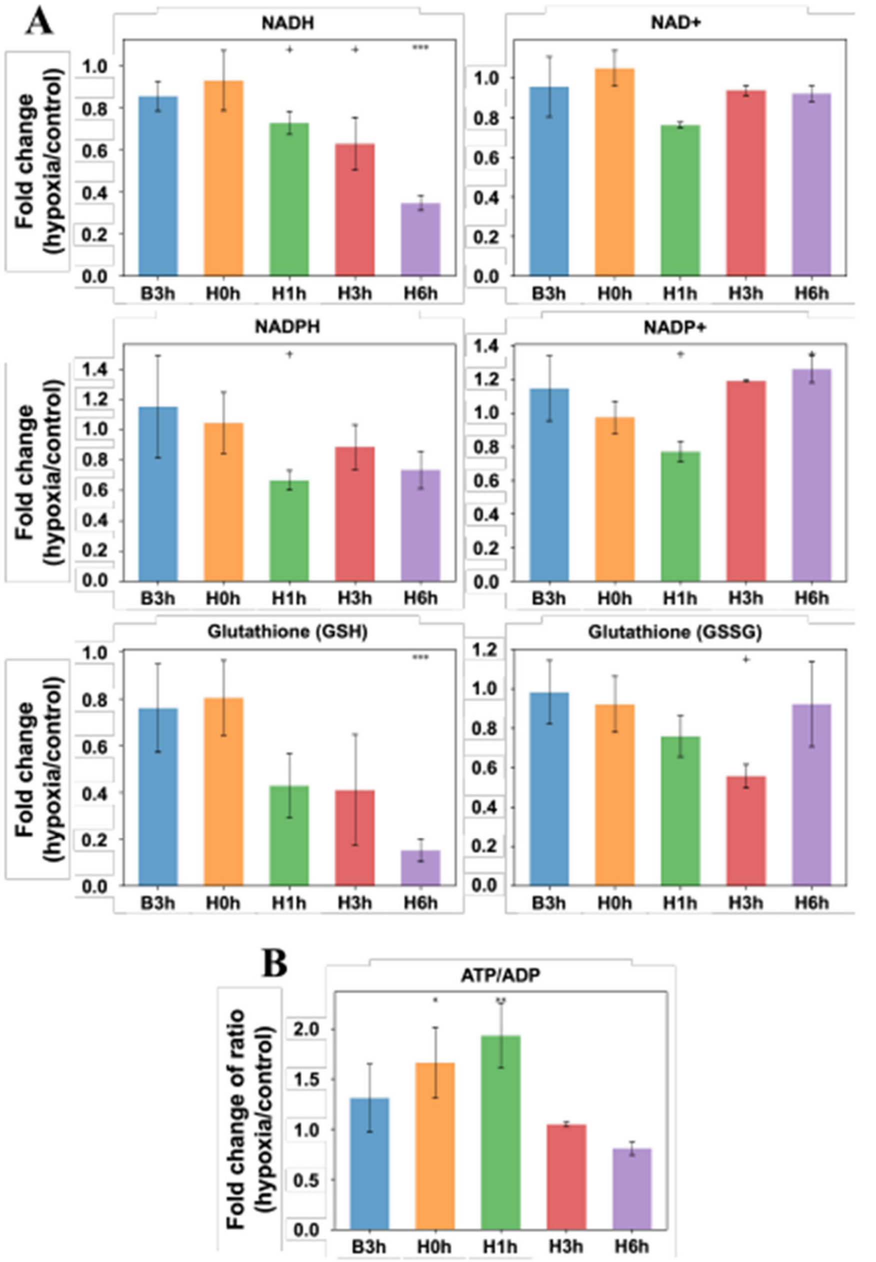

Fig. S12. Relative fold differences of metabolites between hypoxic culture and control culture of AGΔ-GAGΔ. (A) Differences at each timepoint. (B) Ratio of ATP/ADP at each timepoint. Comparisons vs. B3h: +$p < 0.1$, *$p < 0.05$, **$p < 0.01$, ***$p<0.001$. Values were normalized to control at each timepoint.
