# Supplementary material for: Analysis of Short-Term Responses to Hypoxia During Stirred-Tank Fermentation in Aspergillus oryzae
Source: J Fungi (Basel). 2026 May 7;12(5):347. doi: 10.3390/jof12050347 (PMC13208471; doi:10.3390/jof12050347)
Supplement: Supplementary file 1 [file jof-12-00347-s001.zip › supplementary Tables S1-S6.pdf]

Table S1. Strains used in this study.

Strain	Genotype	References
WT	*ΔligD::sC, ΔadeA::ptrA, adeA+, PglaA142-cutL1::niaD*	(Miyazawa et al., 2016; Miyazawa et al., 2019)
AGΔ-GAGΔ	*ΔligD::sC, ΔadeA::ptrA, agsA::loxP, agsB::loxP, agsC::loxP, sphZugeZ::adeA, PglaA142-cutL1::niaD*	(Miyazawa et al., 2019)
AGΔ-GAGΔ-ΔAOXA	*ΔligD::sC, ΔadeA::ptrA, agsA::loxP, agsB::loxP, agsC::loxP, sphZugeZ::loxP, aoxA::adeA, PglaA142-cutL1::niaD*	This study

Table S2. Primers used for the construction of AGΔ-GAGΔ-ΔAOXA.

Purpose	Primer	Sequence
aoxA disruption	AoaoxA-LU-Eco-FW	CGGGTACCGAGCTCGAATTCGAGTCCCGTTACCTAGGAATCGAC
	AoaoxA-LL-RV	GATATACCGTGACTTTTTAGGGGAAGCTACGCCTCTACAG
	AoaoxA-RU-FW	TAGTTTCGTCGAGATACTGCGCCGTGCTGAAGCAAGTTTG
	Aoaox-RL-Eco-RV	AAACGACGGCCAGTGAATTCCGTGGATCTAATGCGCTGACTC
	AoadeA-U-FW	CTAAAAAGTCACGGTATATCATGACCACTG
	AoadeA-L-RV	GCAGTATCTCGACGAAACTACCTAATAAC
Quantitative PCR	AoaoxA-RT-forward	ACGATGCAGCAGTCCATTGC
	AoaoxA-RT-reverse	ACCCGGCGCATCTGTTCTTC

Table S3. Composition of the modified chemically defined medium used for lab-scale fermentation.

Component	Final conc. (g/L)
Glucose	60
K_2HPO_4	10
$NaNO_3$	3
$(NH_4)_2SO_4$	0.35
NaCl	1
$MgSO_4 \cdot 7H_2O$	1
$ZnSO_4 \cdot 7H_2O$	0.0072
$CuSO_4 \cdot 5H_2O$	0.0013
$NiCL_2 \cdot 6H_2O$	0.0003
$MnCL_2 \cdot 4H_2O$	0.0035
$FeSO_4 \cdot 7H_2O$	0.0069

Table S4. Differentially expressed gene (DEG) counts in each strain. Conditions : CTRL, control culture; HYPO, hypoxic culture.

Strain	Condition	Timepoint	DEG count
WT	CTRL vs. HYPO	B3h	0
	CTRL vs. HYPO	H0h	98
	CTRL vs. HYPO	H1h	509
	CTRL vs. HYPO	H3h	451
	CTRL vs. HYPO	H6h	1080
	CTRL	B3h vs. H0h	0
	CTRL	B3h vs. H1h	0
	CTRL	B3h vs. H3h	6
	CTRL	B3h vs. H6h	1148
	HYPO	B3h vs. H0h	191
	HYPO	B3h vs. H1h	659
	HYPO	B3h vs. H3h	603
	HYPO	B3h vs. H6h	763
AGΔ-GAGΔ	CTRL vs. HYPO	B3h	52
	CTRL vs. HYPO	H0h	349
	CTRL vs. HYPO	H1h	1604
	CTRL vs. HYPO	H3h	367
	CTRL vs. HYPO	H6h	261
	CTRL	B3h vs. H0h	0
	CTRL	B3h vs. H1h	0
	CTRL	B3h vs. H3h	135
	CTRL	B3h vs. H6h	1191
	HYPO	B3h vs. H0h	2
	HYPO	B3h vs. H1h	1059
	HYPO	B3h vs. H3h	1504
	HYPO	B3h vs. H6h	2122
AGΔ-GAGΔ-ΔAOXA	HYPO	B3h vs. H0h	0
	HYPO	B3h vs. H1h	748
	HYPO	B3h vs. H3h	1054
	HYPO	B3h vs. H6h	3401

Table S5. Differentially expressed gene (DEG) counts between strains. Conditions : CTRL, control culture; HYPO, hypoxic culture.

Strain	Condition	Timepoint	DEG count
WT vs. AGΔ-GAGΔ	CTRL	B3h	2528
	CTRL	H0h	2608
	CTRL	H1h	2845
	CTRL	H3h	2790
	CTRL	H6h	3730
	HYPO	B3h	3063
	HYPO	H0h	3156
	HYPO	H1h	3347
	HYPO	H3h	3090
	HYPO	H6h	2376
WT vs. AGΔ-GAGΔ-ΔAOXA	HYPO	B3h	4123
	HYPO	H0h	4595
	HYPO	H1h	3894
	HYPO	H3h	5017
	HYPO	H6h	4922
AGΔ-GAGΔ vs. AGΔ-GAGΔ-ΔAOXA	HYPO	B3h	5681
	HYPO	H0h	5663
	HYPO	H1h	5236
	HYPO	H3h	5883
	HYPO	H6h	5643

Table S6. Gene set counts in each strain. Conditions : CTRL, control culture; HYPO, hypoxic culture.

Strain	Condition	Timepoint	Enriched GO term count
WT	CTRL vs. HYPO	B3h	0
	CTRL vs. HYPO	H0h	9
	CTRL vs. HYPO	H1h	9
	CTRL vs. HYPO	H3h	12
	CTRL vs. HYPO	H6h	6
	CTRL	B3h vs. H0h	0
	CTRL	B3h vs. H1h	0
	CTRL	B3h vs. H3h	2
	CTRL	B3h vs. H6h	27
	HYPO	B3h vs. H0h	11
	HYPO	B3h vs. H1h	22
	HYPO	B3h vs. H3h	0
	HYPO	B3h vs. H6h	0
AGΔ-GAGΔ	CTRL vs. HYPO	B3h	22
	CTRL vs. HYPO	H0h	48
	CTRL vs. HYPO	H1h	56
	CTRL vs. HYPO	H3h	15
	CTRL vs. HYPO	H6h	11
	CTRL	B3h vs. H0h	0
	CTRL	B3h vs. H1h	0
	CTRL	B3h vs. H3h	16
	CTRL	B3h vs. H6h	15
	HYPO	B3h vs. H0h	9
	HYPO	B3h vs. H1h	24
	HYPO	B3h vs. H3h	29
	HYPO	B3h vs. H6h	28
AGΔ-GAGΔ-ΔAOXA	HYPO	B3h vs. H0h	0
	HYPO	B3h vs. H1h	22
	HYPO	B3h vs. H3h	4
	HYPO	B3h vs. H6h	37

Table S7. Gene expression of selected genes.

Separated excel file.

Table S8. Annotated gene sets for respiratory components.

Separated excel file.
